# Effectiveness of Self-cut vs Mesh-Kit Titanium-Coated Polypropylene Mesh for Transvaginal Treatment of Severe Pelvic Organ Prolapse: A Multicenter Randomized Noninferiority Clinical Trial

**DOI:** 10.1001/jamanetworkopen.2022.31869

**Published:** 2022-09-16

**Authors:** Juan Chen, Jiajie Yu, Abraham Morse, Guangshi Tao, Jian Gong, Binan Wang, Yuling Wang, Gulina Ababaikeli, Xiangyang Jiang, Peishu Liu, Xiaowei Zhang, Hatiguli Nisier, Ping Wang, Christian Fünfgeld, Kuanhui Huang, Heping Zhang, Xin Sun, Lan Zhu

**Affiliations:** 1 Department of Obstetrics and Gynecology, Peking Union Medical College Hospital, Chinese Academy of Medical Science and Peking Union Medical College, National Clinical Research Center for Obstetric and Gynecologic Diseases, Beijing, China; 2 Chinese Evidence-Based Medicine Center, West China Hospital, Sichuan University, Chengdu, Sichuan, China; 3 Department of Obstetrics and Gynecology, Tufts University School of Medicine, Boston, Massachusetts; 4 Department of Obstetrics and Gynecology, The Second Xiangya Hospital of Central South University, Changsha, Hunan, China; 5 Department of Obstetrics and Gynecology, Wuxi Maternal and Child Health Care Hospital, Wuxi, Jiangsu, China; 6 Department of Obstetrics and Gynecology, Changsha Maternal and Child Health Care Hospital, Changsha, Hunan, China; 7 Department of Gynecology, Foshan Women and Children Hospital, Southern Medical University, Foshan, Guangdong, China; 8 Department of Obstetrics and Gynecology, The First Affiliated Hospital of Xinjiang Medical University, Ürümqi, Xinjiang, China; 9 Department of Obstetrics and Gynecology, Shaanxi Provincial People’s Hospital, Xi'an, Shaanxi, China; 10 Department of Obstetrics and Gynecology, Qilu Hospital of Shandong University, Jinan, Shandong, China; 11 Department of Obstetrics and Gynecology, The First Affiliated Hospital of Guangzhou Medical University, Guangzhou, Guangdong, China; 12 Department of Gynecology, The People's Hospital of Xinjiang, Uygur, Autonomous Region Ürümqi15, Xinjiang, China; 13 Department of Gynecology, West China Second Hospital of Sichuan University, Chengdu, Sichuan, China; 14 Key Laboratory of Birth Defects and Related Diseases of Women and Children, Ministry of Education, West China Second Hospital of Sichuan University, Chengdu, Sichuan, China; 15 Department of Obstetrics and Gynecology, Klinik Tettnang, Tettnang, Germany; 16 Department of Obstetrics and Gynecology, Kaohsiung Chang Gung Memorial Hospital, Kaohsiung City, Taiwan; 17 Department of Obstetrics and Gynecology, Xiamen Chang Gung Memorial Hospital, Xiamen, Fujian, China; 18 Department of Biostatistics, Yale University School of Public Health, New Haven, Connecticut; 19 NMPA Key Laboratory for Real World Data Research and Evaluation, Hainan, Chengdu, China; 20 Sichuan Center of Technology Innovation for Real World Data, Chengdu, China

## Abstract

**Importance:**

Transvaginal mesh (TVM) can increase the durability of vaginal surgical procedures for pelvic organ prolapse (POP) and may be indicated in certain situations despite concerns about mesh-related complications. In addition, the expense of commercial mesh kits has limited their use. The effectiveness, safety, and cost of a self-cut mesh procedure compared with a commercial mesh-kit procedure for the surgical treatment of women with POP is unclear.

**Objective:**

To assess the 1-year effectiveness and safety of self-cut titanium-coated polypropylene mesh compared with a precut commercial mesh kit for the transvaginal surgical treatment of women with severe symptomatic POP.

**Design, Setting, and Participants:**

This multicenter randomized noninferiority clinical trial was conducted at 11 hospitals in 8 provinces of China. A total of 336 women with symptomatic stage 3 to 4 POP were enrolled between January 22, 2018, and November 11, 2019, with follow-up through December 11, 2020.

**Interventions:**

Participants were randomized to receive a TVM procedure using either self-cut mesh (self-cut mesh group) or a precut commercial mesh kit (mesh-kit group), both of which used the same titanium-coated polypropylene mesh.

**Main Outcomes and Measures:**

The primary outcome measure was composite surgical success at 1 year, which was defined as the absence of vaginal bulge symptoms, no additional retreatment for POP, and no vaginal prolapse at or beyond the hymen. Secondary outcomes included symptom-specific pelvic floor function and quality-of-life measures as well as perioperative complications, including mesh-related complications and hospitalization costs. Complications were categorized using the Clavien-Dindo system (with grade 1 indicating any deviation from the normal postoperative course but not requiring grade 2-4 interventions; grade 2, need for pharmacological treatment, blood transfusion, and/or total parenteral nutrition; grade 3, the need for surgical, endoscopic, and/or interventional radiological procedures; and grade 4, life threatening).

**Results:**

Among 336 female participants (mean [SD] age, 63.3 [5.9] years; all of Chinese ethnicity), 169 patients were randomized to the self-cut mesh group, and 167 were randomized to the mesh-kit group. Three patients were unavailable for follow-up after 1 year. In the intention-to-treat analysis, 162 women (95.9%) in the self-cut mesh group had outcomes that met the definition of surgical success; this result was noninferior to the surgical success rate observed in the mesh-kit group (146 women [87.4%]; risk difference, 8.5%; 95% CI, 2.2%-14.3%; *P* = .006). The frequency of Clavien-Dindo grade 1 to 3 perioperative complications was not significant between groups (12 of 166 women [7.2%] in the self-cut mesh group vs 20 of 161 women [12.4%] in the mesh-kit group; *P* = .14). Vaginal mesh exposure rates in women examined at 1 year were similar (4 women [2.4%] in the self-cut mesh group vs 8 women [4.8%] in the mesh-kit group; *P* = .23). Median (IQR) total hospitalization costs were $3663.00 ($3258.90-$4495.10) in the self-cut mesh group vs $6144.00 ($5434.90-$7160.20) in the mesh-kit group (*P* < .01), representing savings of $2481.00 (40.4%) with the use of self-cut mesh.

**Conclusions and Relevance:**

In this clinical trial, the composite surgical success rate of a self-cut mesh procedure was noninferior to that of a commercial mesh-kit procedure using the same titanium-coated polypropylene mesh and reduced hospitalization expenses by 40.4%. These findings suggest that the use of self-cut mesh procedures may be advantageous for the surgical treatment of some women with severe POP, particularly those in countries with low and middle income.

**Trial Registration:**

ClinicalTrials.gov identifier: NCT03283124

## Key Points

**Question:**

Is a self-cut transvaginal mesh procedure vs a commercial mesh-kit procedure effective and safe for the treatment of women with symptomatic pelvic organ prolapse?

**Findings:**

In this randomized clinical trial including 336 women undergoing transvaginal mesh procedures, 95.9% of women in the self-cut mesh group experienced outcomes that met the definition of composite success, which was statistically noninferior to the 87.4% surgical success rate in the mesh-kit group. The self-cut mesh procedure also reduced hospitalization costs by 40.4%.

**Meaning:**

This study found that the use of self-cut mesh was effective, safe, and less expensive than a commercial mesh kit for the transvaginal surgical treatment of women with pelvic organ prolapse, suggesting that self-cut mesh procedures may be advantageous for some patients in countries with low and middle income.

## Teaser

This randomized clinical trial assesses the 1-year effectiveness and safety of self-cut vs mesh-kit titanium-coated polypropylene mesh for the transvaginal surgical treatment of women with severe pelvic organ prolapse.

## Introduction

Pelvic organ prolapse (POP) is a common health problem that can have substantial adverse effects on a woman’s health and quality of life. The prevalence of symptomatic POP in China is 9.6% according to 1 national study published in 2021.^1^ Although transvaginal mesh (TVM) procedures are still associated with some controversy, they can increase the durability of vaginal surgical procedures for pelvic organ prolapse (POP) and may be indicated in certain situations despite concerns about mesh-related complications. National guidelines in China include the recommendation that vaginal mesh may be appropriate to use among patients with severe (Pelvic Organ Prolapse Quantification [POP-Q] system stage 3, indicating the most distal portion of the prolapse protrudes >1 cm below the hymen but no farther than 2 cm less than the total vaginal length, or stage 4, indicating vaginal eversion is essentially complete) or recurrent POP.^2^

Most of the commercially available mesh kits in China are imported, and the high cost (approximately $3000-$4000) poses a substantial challenge to some patients because it is not totally covered by insurance in China. In 2006, a TVM system that included reusable trocars and self-cut mesh, which could reduce cost for patients, was designed.^3^ From 2006 to 2008, a multicenter prospective clinical trial^4^ was conducted to evaluate anatomical and quality-of-life outcomes among those with severe POP who received TVM repair using self-cut mesh. This study found that the anatomical success rate was 91.7%, and there were significant improvements in quality of life; the mesh exposure rate was 6.9%.^4^ (Mesh exposure was defined as vaginal mesh visualized through separated vaginal epithelium.^5^) After 7 years of follow-up, self-cut TVM repair had good long-term results, with 84.3% of patients experiencing continued anatomical success (POP-Q stage 0, indicating no prolapse, or stage 1, indicating the most distal portion of the prolapse was >1 cm above the level of the hymen) and 8.9% of patients having mesh-related complications.^6^

The primary goal of this randomized noninferiority clinical trial was to investigate whether self-cut mesh was as effective and safe as a precut commercial mesh kit for TVM surgical procedures among patients with severe POP. We also used the opportunity to calculate the total mean hospital costs of TVM procedures using self-cut vs mesh-kit titanium-coated polypropylene mesh.

## Methods

### Study Design and Oversight

Details of the design of this randomized clinical trial were published previously.^7^ The study was approved by the institutional review board of Peking Union Medical College Hospital. The trial protocol and statistical analysis plan are available in Supplement 1. Clinical trial oversight and monitoring were provided by a steering committee and an independent data and safety monitoring board whose members reviewed safety data during the study period (eAppendix in Supplement 2). All participants provided written informed consent. This study followed the Consolidated Standards of Reporting Trials (CONSORT) reporting guideline for randomized clinical trials.

### Participants

Participants were recruited at 11 hospitals in 8 provinces of China (eTable 1 in Supplement 2) and enrolled between January 22, 2018, and November 11, 2019, with follow-up through December 11, 2020. To minimize performance bias, only surgeons with adequate experience in TVM procedures (>20 cases per year) and the ability to perform both procedures served as primary surgeons in this clinical trial. Patients with symptomatic POP were eligible for enrollment if they presented with POP-Q stages 3 to 4 and had only mild to moderate posterior prolapse (defined as point B anterior [Ba] and/or cervix or vaginal cuff [C] >1 cm and point B posterior [Bp] ≤1 cm; in the POP-Q system, each measurement point is measured in centimeters above or proximal to the hymen [negative number] or in centimeters below or distal to the hymen [positive number], with the plane of the hymen defined as 0). Women were eligible for inclusion if they experienced menopause more than 3 years before enrollment or were older than 55 years. All participants chose to undergo TVM treatment after appropriate surgical counseling and before any discussion of study participation, and all participants consented that they were able to adhere to the follow-up regimen. Women were excluded if they were older than 75 years; had high surgical risk due to medical comorbidities, such as active gynecological cancer; had severe cardiovascular or respiratory disease; and/or were recommended to receive a concomitant anti-incontinence procedure.

### Randomization

After providing informed consent, patients were enrolled in a web-based electronic data capture system by a research staff member and assigned a unique study number before randomization. Patients were randomized on a 1:1 ratio to either the self-cut mesh group or the mesh-kit group according to a central randomization system with a block size of 6. Randomization was stratified by center. The patient and surgeon were informed about the allocated operative procedure after randomization.

Details of the surgical procedures were published previously.^3^ In brief, the self-cut mesh procedure involved a single piece of titanium-coated polypropylene mesh (TiLOOP, 10 cm × 15 cm; pfm medical) that was cut into 2 pieces for the anterior and apical compartment reconstructions. The superficial anterior arm penetrated through the obturator at approximately 1 cm from the proximal (prepubic) end of the arcus tendinous fasciae pelvis, and the deep anterior arm penetrated the middle of the arcus tendinous fasciae pelvis. The mesh strips went through bilateral ischial spine fascia, and the ends of the strips were fixed to the uterosacral ligament. We used traditional posterior colporrhaphy to repair the distal two-thirds of the posterior vaginal wall.

The surgical procedure using the commercially available titanium-coated polypropylene mesh kit (TiLOOP Total 6; pfm medical) involved insertion of the mesh with provided tunnelers for the transobturator and ischiorectal passage. No separate posterior colporrhaphy was performed (eFigure 1 in Supplement 2).

### Outcome Measures

The primary outcome was the composite surgical success rate at 1 year after the TVM procedure. The definition of composite surgical success included (1) the absence of vaginal bulge symptoms, as indicated by a rating of 0 on question 3 of the 20-item Pelvic Floor Distress Inventory (PFDI-20), which stated, “Do you usually have a bulge or something falling out that you can see or feel in your vaginal area?”; (2) no additional retreatment (surgical procedure or pessary) for POP; and (3) no POP-Q point at or beyond the hymen.

Secondary outcomes included anatomical outcomes (individual compartment POP-Q point measurements), symptom improvement, perioperative parameters, perioperative and 1-year complications, and cost. Perioperative complications were scored according to the Clavien-Dindo classification system,^8^ with grade 1 defined as any deviation from the normal postoperative course but not requiring grade 2 to 4 interventions; grade 2, need for pharmacological treatment, blood transfusion, and/or total parenteral nutrition as a result of the complication; grade 3, need for surgical, endoscopic, and/or interventional radiological procedures; and grade 4, life-threatening complication. Cost calculation included only direct hospitalization expenses, including costs of prescription drugs, laboratory and radiological procedures, surgical procedures and anesthesia, and materials (eg, surgical mesh).

After receiving the procedure, patients were followed up at 3 months and 1 year. A staff physician or research nurse who was not involved in the patient’s treatment and was blinded to the patient’s group assignment performed the pelvic examination and questionnaire data collection. Questionnaires included the Chinese versions of the 7-item Pelvic Floor Impact Questionnaire (PFIQ-7; comprising subscales from the Colorectal-Anal Impact Questionnaire, the Pelvic Organ Prolapse Impact Questionnaire, and the Urinary Impact Questionnaire; the total PFIQ-7 score is the sum of the mean scores of the 3 subscales [range, 0-100 points each], with summary scores ranging from 0-300 points and higher scores indicating greater symptom severity)^9^ and the PFDI-20 (comprising subscales from the Colorectal-Anal Distress Inventory, the Pelvic Organ Prolapse Distress Inventory, and the Urinary Distress Inventory; the total PFDI-20 score is the sum of the mean scores of the 3 subscales [range, 0-100 points each], with summary scores ranging from 0-300 points and higher scores indicating worse quality of life).^10^ For sexually active women, the Chinese version of the 12-item Pelvic Organ Prolapse/Urinary Incontinence Sexual Questionnaire (PISQ-12; score range, 0-48 points, with higher scores indicating better sexual function) was administered.^11^ The Patient Global Impression of Improvement scale (comprising 1 question that asks patients to rate their urinary tract conditions now vs before beginning treatment on a scale of 1-7, with 1 indicating very much better and 7 indicating very much worse)^12^ was used to assess women’s perceptions of improvement. Mesh-related complications were categorized using the International Urogynecological Association/International Continence Society joint terminology coding system (comprising category, time, and site).^5^

### Statistical Analysis

According to a previous study by Fünfgeld et al^13^ that used the same commercial mesh kit (TiLOOP Total 6) as the current study, the anatomical success rate after 12 months across all compartments was 86%. Based on an estimated success rate of 90% for the current study and using 10% as the noninferiority margin (β = 0.2; 1-sided α = .025), 284 patients (142 in each group) were required. Assuming that 10% of participants might not continue participation to the 1-year follow-up visit, a goal of 316 patients was set for recruitment, and 336 patients were enrolled.

We used an independent sample *t* test or nonparametric Mann-Whitney *U* test for continuous outcomes and a Fisher exact test or χ^2^ test for categorical outcomes. We used a paired samples *t* test to compare mean continuous data within groups. For anatomical (POP-Q point measurements) and symptom improvement outcomes, we compared the changes from baseline to 1 year of follow-up. All estimated differences (with 95% CIs) for the primary outcome were reported; the Agresti-Coull method^14^ was used to calculate 95% CIs for differences in proportions. No adjustment for multiple comparisons was performed for any of the analyses, and 2-tailed *P* < .05 was considered significant for secondary outcomes. For primary outcome noninferiority measures, statistical significance was set at 1-sided α = .025. Significant *P* values indicated noninferiority (ie, the upper limit of the 95% CI of the between-group difference exceeded the noninferiority threshold of 10%).

All analyses were performed according to the intention-to-treat (ITT) principle, which included all patients randomized to receive an allocated procedure, irrespective of whether the actual procedure was performed. We applied multiple imputation techniques (based on baseline covariates and treatment group) for all women with missing data on the primary outcome at 12 months and calculated pooling effects of 5 imputed databases. Robustness of the primary outcome was evaluated in sensitivity analyses of the ITT population without imputation (eTable 2 in Supplement 2). Three subgroup analyses (body mass index [calculated as weight in kilograms divided by height in meters squared], history of POP procedure, and POP-Q stage) of the primary outcomes were performed among the ITT population to explore subgroup effects (eFigure 2 in Supplement 2). All statistical analyses were performed using SPSS Statistics software, version 24.0 (IBM) for Windows (Microsoft Corporation).

## Results

### Participants

Between January 2018 and November 2019, 336 women (mean [SD] age, 63.3 [5.9] years; all of Chinese ethnicity) were randomized to either the self-cut mesh group (n = 169) or the mesh-kit group (n = 167) (Figure). There were no significant differences between the 2 groups at baseline (Table 1). For example, in the self-cut mesh group vs the mesh-kit group, the mean (SD) body mass index was 24.2 (2.8) vs 24.2 (2.5). Six patients (3.6%) in the self-cut mesh group vs 9 patients (5.4%) in the mesh-kit group received a previous POP procedure. Most patients had POP-Q stage 3 (113 women [66.9%] in the self-cut mesh group vs 121 women [72.5%] in the mesh-kit group). Of 29 patients with missing POP-Q point measurements at 1 year of follow-up, 7 had complete 2-year follow-up data, and we imputed the primary outcome for these patients using the 2-year data. Three patients were unavailable for follow-up.

**Figure.**
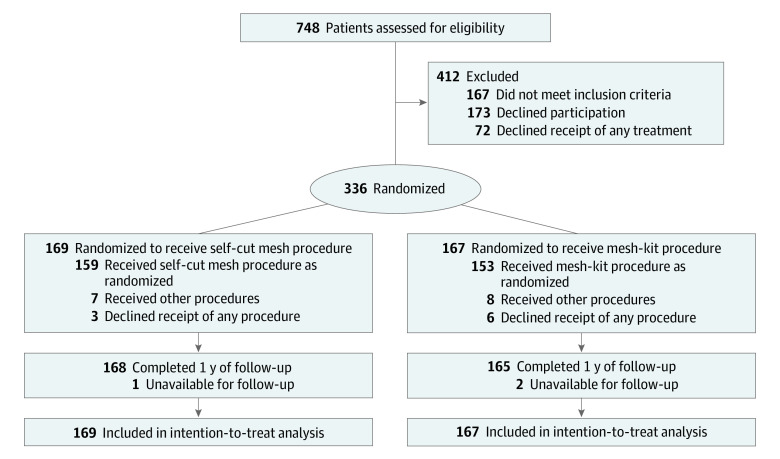
Study Flowchart

**Table 1.** Baseline Characteristics of Intention-to-Treat Population^a^

Characteristic	Patients, No. (%)	
	Self-cut mesh group (n = 169)	Mesh-kit group (n = 167)
Age, mean (SD), y	63.8 (5.8)	62.8 (5.9)
BMI, mean (SD)	24.2 (2.8)	24.2 (2.5)
Parity, median (IQR)	2 (1-3)	2 (1-3)
Current smoker	1 (0.6)	0
Postmenopausal	167 (98.8)	165 (98.8)
Time since menopause, median (IQR), y	14 (8-18)	13 (9-18)
Hormone therapy	1 (0.6)	2 (1.2)
Previous POP procedure	6 (3.6)	9 (5.4)
Previous stress urinary incontinence procedure	1 (0.6)	0
Other previous pelvic procedure	38 (22.5)	51 (30.5)
Posthysterectomy	10 (5.9)	20 (12.0)
Comorbidities		
Diabetes	22 (13.0)	28 (16.8)
Chronic bronchitis	8 (4.7)	3 (1.8)
Constipation	23 (13.6)	17 (10.2)
Coronary heart disease	7 (4.1)	6 (3.6)
Hypertension	60 (35.5)	69 (41.3)
POP-Q stage^b^		
3	113 (66.9)	121 (72.5)
4	56 (33.1)	46 (27.5)
POP-Q point measurement, mean (SD), cm^c^		
Aa	1.8 (1.0)	1.7 (1.0)
Ap	–0.9 (1.0)	–0.9 (1.0)
Ba	4.1 (1.3)	4.1 (1.2)
Bp	–0.6 (1.2)	–0.7 (1.0)
C	3.0 (2.6)	2.7 (2.5)
GH	4.9 (1.2)	4.4 (1.4)
PB	2.5 (0.9)	2.4 (0.9)
TVL	7.6 (0.8)	7.5 (0.8)
Maximum flow rate, median (IQR), mL/s	22.2 (16.0-30.2)	20.8 (13.8-29.3)
Mean flow rate, median (IQR), mL/s	10.7 (7.2-16.2)	9.6 (6.7 to 14.2)
MCC, median (IQR), mL	393.7 (341.0-489.0)	408.5 (324.5-512.1)
Postvoid residual, median (IQR), mL	0 (0-22)	0 (0-45)
Urinary incontinence		
Stress	21 (12.4)	16 (9.6)
Urge	2 (1.2)	2 (1.2)
Mixed	4 (2.4)	7 (4.2)
Patient-reported outcome scores, median (IQR)		
PFDI-20^d^	70.8 (39.1-108.3)	70.8 (45.8-104.2)
CRADI	6.2 (0-15.6)	6.2 (0-15.6)
POPDI	37.5 (25.0-54.2)	37.5 (20.8-54.2)
UDI	20.8 (8.3-41.7)	25.0 (12.5-41.7)
PFIQ-7^e^	66.7 (28.6-100)	52.4 (28.6-85.7)
CRAIQ	0 (0-11.9)	0 (0-4.8)
POPIQ	38.1 (23.8-57.1)	33.3 (19.0-52.4)
UIQ	19.0 (0-42.8)	19.0 (0-38.1)
Sexually active	51 (30.2)	43 (25.7)
PISQ-12 score among sexually active women, mean (SD)^f^	27.5 (5.9)	27.8 (5.9)

Abbreviations: BMI, body mass index (calculated as weight in kilograms divided by height in meters squared); MCC, maximum cystometric capacity; POP, pelvic organ prolapse; TVL, total vaginal length.

^a^ There were no statistically significant differences in baseline characteristics between the randomized groups.

^b^ In the Pelvic Organ Prolapse Quantification (POP-Q) system, stage 0 indicates no prolapse; stage 1, the most distal portion of the prolapse is >1 cm above the level of the hymen; stage 2, the most distal portion of the prolapse is ≤1 cm proximal or distal to the plane of the hymen; stage 3, the most distal portion of the prolapse protrudes >1 cm below the hymen but no farther than 2 cm less than the TVL; and stage 4, vaginal eversion is essentially complete.

^c^ The POP-Q system comprises 6 points of measurement (A anterior [Aa], A posterior [Ap], B anterior [Ba], B posterior [Bp], cervix or vaginal cuff [C], and posterior fornix [D; omitted after total hysterectomy and not measured in this study]) plus 3 additional measurements (genital hiatus [GH], perineal body [PB], and TVL). The hymen is the fixed point of reference; each point is measured in centimeters above or proximal to the hymen (negative number) or in centimeters below or distal to the hymen (positive number), with the plane of the hymen defined as 0.

^d^ The 20-item Pelvic Floor Distress Inventory (PFDI-20) comprises subscales from the Colorectal-Anal Distress Inventory (CRADI), the Pelvic Organ Prolapse Distress Inventory (POPDI), and the Urinary Distress Inventory (UDI). The total PFDI-20 score is the sum of the mean scores of the 3 subscales (range, 0-100 points each), with summary scores ranging from 0 to 300 points and higher scores indicating worse quality of life.

^e^ The 7-item Pelvic Floor Impact Questionnaire (PFIQ-7) comprises subscales from the Colorectal-Anal Impact Questionnaire (CRAIQ), the Pelvic Organ Prolapse Impact Questionnaire (POPIQ), and the Urinary Impact Questionnaire (UIQ). The total PFIQ-7 score is the sum of the mean scores of the 3 subscales (range, 0-100 points each), with summary scores ranging from 0 to 300 points and higher scores indicating greater symptom severity.

^f^ Scores on the 12-item Pelvic Organ Prolapse/Urinary Incontinence Sexual Questionnaire (PISQ-12) range from 0 to 48 points, with higher scores indicating better sexual function.

### Primary Outcome

Results of the analyses of the primary outcome and anatomical compartments at 1 year are shown in Table 2. In the ITT analysis, the composite success rate in the self-cut mesh group was 95.9% (162 patients), which was noninferior to the composite success rate of 87.4% (146 patients) in the mesh-kit group, representing a risk difference of 8.5% (95% CI, 2.2%-14.3%; *P* = .006). The results of the sensitivity analyses were consistent with those of the ITT analysis (eTable 2 and eFigure 2 in Supplement 2).

**Table 2.** Primary and Anatomical Outcomes at 1 Year in Intention-to-Treat Population

Outcome	Patients, No. (%)		Risk difference, % (95% CI)	*P* value
	Self-cut mesh group (n = 169)	Mesh-kit group (n = 167)		
Composite success rate	162 (95.9)	146 (87.4)	8.5 (2.2 to 14.3)	.006
Component success rates				
Symptom improvement	162 (95.9)	153 (91.6)	4.3 (–1.3 to 9.6)	.11
Anatomical	162 (95.9)	150 (89.8)	6.1 (–1.2 to 10.7)	.04
Repeat procedure for recurrence	0	2 (1.2)	–1.2 (–4.0 to 1.6)	.25
Anatomical failure at 1-y visit	6 (3.6)	13 (7.8)	–4.2 (–9.2 to 0.7)	.09
Cumulative anatomical failure at 1 y				
Anterior compartment	3 (1.8)	3 (1.8)	0 (–3.6 to 3.5)	>.99
Apical compartment	2 (1.2)	0	1.2 (–1.6 to 3.9)	.50
Posterior compartment	1 (0.6)	11 (6.6)	–6.0 (–1.4 to –10.3)	.003
All compartments	0	1 (0.6)	–0.6 (–3.1 to 1.9)	.50

For the components of the composite success rate, 162 women (95.9%) in the self-cut mesh group experienced symptom improvement compared with 153 women (91.6%) in the mesh-kit group (risk difference, 4.3%; 95% CI, −1.3% to 9.6%; *P* = .11), and 162 women (95.9%) in the self-cut mesh group experienced anatomical success compared with 150 women (89.8%) in the mesh-kit group (risk difference, 6.1%; 95% CI, −1.2% to 10.7%; *P* = .04); however, neither difference was statistically significant. Two women in the mesh-kit group declined receipt of a pessary and underwent colpocleisis for recurrence within 1 year after the index procedure.

Anatomical failure (defined as any POP-Q point at or beyond the hymen) occurred in 6 women (3.6%) in the self-cut mesh group compared with 13 women (7.8%) in the mesh-kit group (risk difference, −4.2%; 95% CI, −9.2% to 0.7%; *P* = .09). No significant differences were found for anatomical failure in the anterior compartment (3 women [1.8%] in both groups; risk difference, 0%; 95% CI, −3.6% to 3.5%; *P* > .99), the apical compartment (2 women [1.2%] in the self-cut mesh group vs 0 women in the mesh-kit group; risk difference, 1.2%; 95% CI, −1.6% to 3.9%; *P* = .50), or all compartments (0 women vs 1 woman [0.6%] in the mesh-kit group; risk difference, −0.6%; 95% CI, −3.1% to 1.9%; *P* = .50). However, a significant difference was observed in the posterior compartment, with 1 patient (0.6%) in the self-cut mesh group vs 11 patients (6.6%) in the mesh-kit group (risk difference, −6.0%; 95% CI, –1.4% to –10.3%; *P* = .003) experiencing a recurrence. The overall anatomical failure rate was 3.6% (6 patients) in the self-cut mesh group vs 9.0% (15 patients) in the mesh-kit group. In a sensitivity analysis that assumed all posterior failures in the commercial mesh-kit group would have been avoided with the performance of traditional posterior repair (which was not provided to that group), self-cut mesh remained noninferior to the commercial mesh kit (success rates: 95.9% [162 patients] vs 94.0% [157 patients]; risk difference, 1.9%; 95% CI, –2.9% to 6.5%; *P* < .001).

### Secondary Outcomes

All patients with an intact uterus received either a transvaginal or laparoscopic hysterectomy based on the operating gynecologist’s preference. The reason for hysterectomy was to standardize the intervention. Perioperative parameters of the 2 groups, including number of postoperative hospitalization days and visual analog pain scores, are shown in eTable 3 in Supplement 2. Compared with the mesh-kit group, the self-cut mesh group had longer operative time (mean [SD], 110.7 [48.2] minutes vs 99.7 [49.4] minutes; *P* = .04) and lower estimated blood loss (median [IQR], 100.0 [57.5-100.0] mL vs 100.0 [100.0-150.0] mL; *P* = .03).

With regard to anatomical points, the extent of Ap and Bp improvement was greater in the self-cut mesh group vs the mesh-kit group (Ap: mean [SD], −1.5 [1.1] cm vs −1.1 [1.1] cm; *P* = .007; Bp: mean [SD], −1.8 [1.4] cm vs −1.4 [1.3] cm; *P* = .02) (Table 3). For pelvic floor function, changes in scores among the self-cut mesh vs mesh-kit group differed on the Colorectal-Anal Impact Questionnaire subscale of the PFIQ-7 (median [IQR], 0 [−9.5 to 0] points vs 0 [−4.8 to 0] points; *P* = .04). With regard to quality of life, changes in summary scores on the PFIQ-7 also differed between the self-cut mesh vs mesh-kit group (median [IQR], −52.4 [−95.2 to −28.6] points vs −47.6 [−80.9 to −16.7] points; *P* = .04). There was no significant difference in PISQ-12 scores in the self-cut mesh vs mesh-kit group either before the procedure (mean [SD], 27.5 [5.9] points vs 28.2 [5.8] points; *P* = .83) or after the procedure (mean [SD], 31.4 [6.4] points vs 33.1 [5.0] points; *P* = .23). Median (IQR) total hospitalization costs were $3663.00 ($3258.90-$4495.10) in the self-cut mesh group vs $6144.00 ($5434.90-$7160.20) in the mesh-kit group (*P* < .01), representing savings of $2481.00 (40.4%) with the use of self-cut mesh.

**Table 3.** Secondary Outcomes^a^

Outcome	Change at 1 y		*P* value
	Self-cut mesh group (n = 169)	Mesh-kit group (n = 167)	
Anatomical			
POP-Q point measurement, mean (SD), cm^b^			
Aa	–4.0 (1.2)	–3.9 (1.2)	.39
Ap	–1.5 (1.1)	–1.1 (1.1)	.007
Ba	–6.5 (1.5)	–6.4 (1.4)	.61
Bp	–1.8 (1.4)	–1.4 (1.3)	.02
C	–9.4 (2.8)	–9.1 (2.7)	.34
GH	–0.8 (1.5)	–0.6 (1.5)	.18
PB	0.4 (0.7)	0.5 (0.7)	.70
TVL	–0.5 (0.8)	–0.5 (0.9)	.77
Symptom improvement			
PFDI-20 score, median (IQR)^c^	–53.1 (–93.2 to –18.0)	–50.0 (–81.2 to –24.0)	.57
CRADI	–1.6 (–12.5 to 0)	–3.1 (–9.4 to 0)	.45
POPDI	–33.3 (–50.0 to –16.7)	–29.2 (–50.0 to –14.6)	.22
UDI	–14.6 (–29.2 to –4.2)	–16.7 (–29.2 to –4.2)	.48
PFIQ-7 score, median (IQR)^d^	–52.4 (–95.2 to –28.6)	–47.6 (–80.9 to –16.7)	.04
CRAIQ	0 (–9.5 to 0)	0 (–4.8 to 0)	.04
POPIQ	–33.3 (–52.4 to –19.0)	–28.6 (–47.6 to –9.5)	.05
UIQ	–14.3 (–38.1 to 0)	–14.3 (–33.3 to 0)	.42
PISQ-12 score, mean (SD)^e^			
Before procedure^f^	27.5 (5.9)	28.2 (5.8)	.83
After procedure^g^^,^^h^	31.4 (6.4)	33.1 (5.0)	.23
PGI-I response of *much better* or *very much better*, No. (%)^i^	162 (95.9)	153 (91.6)	.12
Cost, median (IQR), $	3663.00 (3258.90 to 4495.10)	6144.00 (5434.90 to 7160.20)	<.001

^a^ All continuous data were calculated as change from baseline to end of follow-up at 1 year, with the exception of Pelvic Organ Prolapse/Urinary Incontinence Sexual Questionnaire (PISQ-12) score and cost.

^b^ The Pelvic Organ Prolapse Quantification (POP-Q) system comprises 6 points of measurement (A anterior [Aa], A posterior [Ap], B anterior [Ba], B posterior [Bp], cervix or vaginal cuff [C], and posterior fornix [D; omitted after total hysterectomy and not measured in this study]) plus 3 additional measurements (genital hiatus [GH], perineal body [PB], and total vaginal length [TVL]). The hymen is the fixed point of reference; each point is measured in centimeters above or proximal to the hymen (negative number) or in centimeters below or distal to the hymen (positive number), with the plane of the hymen defined as 0.

^c^ The 20-item Pelvic Floor Distress Inventory (PFDI-20) comprises subscales from the Colorectal-Anal Distress Inventory (CRADI), the Pelvic Organ Prolapse Distress Inventory (POPDI), and the Urinary Distress Inventory (UDI). The total PFDI-20 score is the sum of the mean scores of the 3 subscales (range, 0-100 points each), with summary scores ranging from 0 to 300 points and higher scores indicating worse quality of life.

^d^ The 7-item Pelvic Floor Impact Questionnaire (PFIQ-7) comprises subscales from the Colorectal-Anal Impact Questionnaire (CRAIQ), the Pelvic Organ Prolapse Impact Questionnaire (POPIQ), and the Urinary Impact Questionnaire (UIQ). The total PFIQ-7 score is the sum of the mean scores of the 3 subscales (range, 0-100 points each), with summary scores ranging from 0 to 300 points and higher scores indicating greater symptom severity.

^e^ Scores on the 12-item PISQ-12 range from 0 to 48 points, with higher scores indicating better sexual function.

^f^ Includes 51 patients in the self-cut mesh group and 43 patients in the mesh-kit group.

^g^ Includes 31 patients in the self-cut mesh group and 40 patients in the mesh-kit group.

^h^ Paired sample test of PISQ-12 score at baseline and follow-up (self-cut mesh group: *P* = .08; mesh-kit group: *P* < .001).

^i^ The Patient Global Impression of Improvement (PGI-I) comprises 1 question asking patients to rate their urinary tract conditions now vs before beginning treatment on a scale of 1 (very much better) to 7 (very much worse).

### Adverse Events

Clavien-Dindo grade 1 to 3 perioperative complications were reported in 12 of 166 patients (7.2%) in the self-cut mesh group vs 20 of 161 patients (12.4%) in the mesh-kit group (*P* = .14) (Table 4). Delayed voiding, defined as more than 100 mL of urine retention after removal of the catheter, was similar between the groups (4 of 166 patients [2.4%] in the self-cut mesh group vs 8 of 161 patients [5.0%] in the mesh-kit group). Most perioperative complications were classified as Clavien-Dindo grades 1 to 2, and they occurred in 10 of 166 patients (6.0%) in the self-cut mesh group vs 18 of 161 patients (11.2%) in the mesh-kit group and required only conservative treatment. Four Clavien-Dindo grade 3b complications (indicating bladder injury during the procedure) were repaired during the procedures without sequelae.

**Table 4.** Safety Data

Complication	Patients, No./total No. (%)	
	Self-cut mesh group	Mesh-kit group
Perioperative^a^	12/166 (7.2)	20/161 (12.4)
Bladder injury	2/166 (1.2)	2/161 (1.2)
Hematoma	3/166 (1.8)	5/161 (3.1)
Blood transfusion	0	1/161 (0.6)
Infection requiring antibiotic treatment	1/166 (0.6)	5/161 (3.1)
Cerebrovascular accident	1/166 (0.6)	1/161 (0.6)
Intermuscular venous thrombosis	2/166 (1.2)	1/161 (0.6)
Delayed voiding	4/166 (2.4)	8/161 (5.0)
Clavien–Dindo grade^b^		
1-2	10/166 (6.0)	18/161 (11.2)
3	2/166 (1.2)	2/161 (1.2)
At 1 y^c^		
De novo defecation	2/169 (1.2)	4/167 (2.4)
De novo dyspareunia	2/169 (1.2)	1/167 (0.6)
De novo urge urinary incontinence	1/169 (0.6)	0
De novo mixed urinary incontinence	0	4/167 (2.4)
Worsening mixed urinary incontinence	0	1/167 (0.6)
De novo stress urinary incontinence	2/169 (1.2)	3/167 (1.8)
Anti-incontinence procedure	0	3/167 (1.8)
Recurrent urinary infection	0	3/167 (1.8)
Postoperative pain	1/169 (0.6)	2/167 (1.2)
Mesh exposure	4/169 (2.4)	8/167 (4.8)
In-office mesh trimming	3/169 (1.8)	3/167 (1.8)
Operating room mesh trimming	1/169 (0.6)	2/167 (1.2)
Topical estrogen	0	3/167 (1.8)
Local incision scar resection	0	1/167 (0.6)

^a^ Perioperative complications were measured only among participants who received surgical procedures.

^b^ Clavien-Dindo grade 1 was defined as any deviation from the normal postoperative course but not requiring grade 2 to 4 interventions; grade 2, need for pharmacological treatment, blood transfusion, and/or total parenteral nutrition as a result of the complication; grade 3, need for surgical, endoscopic, and/or interventional radiological procedures; and grade 4, life-threatening complication.

^c^ Complications at 1 year of follow-up were measured among all participants.

Among all 336 patients, the vaginal mesh exposure rate within 1 year was 3.6% (12 women), and exposure rates were similar in the 2 groups (4 of 169 patients [2.4%] in the self-cut mesh group vs 8 of 167 patients [4.8%] in the mesh-kit group; *P* = .23). Overall, 3 of 336 patients (0.9%) underwent vaginal mesh trimming in the operating room after in-office trimming failure. Two patients (0.6%) with de novo stress urinary incontinence and 1 patient (0.3%) with worsening mixed urinary incontinence received midurethral sling procedures within 1 year. Three patients (0.9%) experienced de novo chronic pelvic pain. One patient (0.3%) in the mesh-kit group developed severe perineal pain and inflammation around 1 puncture site (International Urogynecological Association/International Continence Society category 6Bb [symptomatic with provoked pain only], T3 [2-12 months after the procedure], S3 [trocar passage]). After ineffective antibiotic treatment and physical therapy, we performed local incision and scar resection for chronic pain. Before scar resection, the patient’s visual analog pain score was 8 to 9; at 3 months after the procedure, the pain had completely resolved.

## Discussion

In this randomized clinical trial, the use of self-cut mesh was noninferior to a commercial mesh kit as a composite measure of TVM surgical success. Several models with imputation for missing data did not differ in results. We found no notable differences in overall anatomical failure, PFDI-20 summary and subscale scores, complications, postoperative recovery, length of hospital stay, and sexual functioning between patients receiving the 2 interventions. The hospital cost of the self-cut mesh group was 40.4% lower than that of the mesh-kit group, owing primarily to the difference in cost between the self-cut mesh and commercial mesh-kit methods.

Some evidence currently supports offering TVM procedures after appropriate patient counseling to women with symptomatic recurrence or a high risk of recurrence (eg, recurrent prolapse, obesity, or large anterior wall defects). Both commercial mesh-kit and self-cut mesh procedures are currently performed in China and other regions in Asia.^15,16,17^ However, there are few studies comparing the use of self-cut materials with precut mesh kits. One study^18^ found similar rates of prolapse recurrence and mesh exposure based on a meta-analysis of 18 studies. Our clinical trial demonstrated that the mesh exposure rate was similar between the 2 groups (2.4% in the self-cut mesh group vs 4.8% in the mesh-kit group).

According to a case series study of a titanium-coated polypropylene mesh kit conducted by Fünfgeld et al,^13^ at 1-year of follow-up, the anatomical recurrence rate was 2.4% for the anterior compartment, 10.1% for the posterior compartment, and 2.8% for the apical compartment. In our clinical trial, the recurrence rate for the mesh-kit group was 1.8% for the anterior compartment, 6.6% for the posterior compartment, 0% for the apical compartment, and 0.6% for all compartments; the overall anatomical failure rate in the mesh-kit group was 9.0%, which was close to our prestudy estimate. In the current study, a standard posterior colporrhaphy was performed in the self-cut mesh group, whereas no posterior colporrhaphy was performed in the mesh-kit group.

Although TVM implantation has the advantage of lower anatomical recurrence rates compared with native tissue repair, it is accompanied by distinct complications, such as mesh exposure, infection, and postoperative pain. In our study, only 12 patients (3.6%) had mesh exposure, and all cases were eventually resolved without sequelae. Three patients (0.9%) had de novo chronic pelvic pain, and all experienced improvement after conservative or surgical treatment.

### Strengths and Limitations

This study has strengths. A major strength is the study’s randomized multicenter design and adequate sample size. Another strength is the performance of blinded postoperative assessments and adjudication of adverse events. In addition, we used questionnaires that were validated among Chinese individuals.

The study also has limitations. First, our findings are based on a relatively short follow-up period of 12 months. Second, patients were not blinded to their randomized treatment because they were required to pay for the self-cut mesh or mesh-kit materials. Third, cost calculations were limited to direct hospital costs only. Fourth, in the mesh-kit group, we did not perform posterior wall repair. At the time this study was designed, we believed that, in the commercial mesh-kit procedure, the ischiorectal passage of the arms that were placed along the full length of the posterior wall and through the sacrospinous ligaments provided more substantive support of the posterior vaginal wall than attachment of the strips to the fascia of the ischial spines that was part of the self-cut mesh procedure. Therefore, we decided to require traditional posterior colporrhaphy in the self-cut mesh group in an effort to equalize this inherent difference in posterior wall support between the 2 procedures. We also decided not to modify the self-cut mesh procedure itself because modification would have decreased our ability to compare the results of this cohort with previous and subsequent results of existing cohorts.

We recognize, in hindsight, that the results of this first direct comparison suggest that our assumption about differential posterior support was not correct. As a result, since the unblinding of our clinical trial data, we no longer exclude traditional posterior repair when performing a commercial mesh-kit procedure. However, when a sensitivity analysis was performed based on the assumption that 100% of the posterior failures in the commercial mesh-kit group would have been avoided with the addition of traditional posterior repair, the findings did not change the conclusion that self-cut mesh was noninferior to the commercial mesh kit (success rate of 95.9% in the self-cut mesh group vs 94.0% in the mesh-kit group; risk difference, 1.9%; 95% CI, –2.9% to 6.5%; *P* < .001).

Due to well-recognized safety concerns and the lack of evidence of additional benefit from TVM procedures, Australia, New Zealand, Scotland, and the United Kingdom have restricted the use or discontinued the marketing of TVM products.^19^ The US Food and Drug Administration ordered manufacturers of TVM kits to discontinue sales and distribution in 2019.^20^ In Asia and some European countries, TVM is still used.^19^ Lightweight macroporous polypropylene mesh appears to have advantages for POP repair based on some evidence of lower complication rates.^21^ However, randomized clinical trials with long-term follow-up to evaluate the effectiveness and safety of lightweight macroporous polypropylene mesh, including the titanium-coated mesh used in this clinical trial, are lacking. The results of this clinical trial provide more evidence to support the continued use of mesh for specific indications and with appropriate counseling in countries where mesh kits remain available.

## Conclusions

This randomized clinical trial demonstrated that the composite success rate of a TVM procedure using self-cut titanium-coated polypropylene mesh was noninferior to a precut commercial kit using the same mesh. The total hospital cost of treatment in the self-cut mesh group was lower than that of the mesh-kit group, suggesting that the use of self-cut mesh procedures may be advantageous for the surgical treatment of some women with severe POP, particularly those in countries with low and middle income.

## Notes

Supplement 1.
Trial Protocol and Statistical Analysis Plan

Supplement 2. **eFigure 1.** Side-by-Side Images of Self-cut Mesh and Mesh Kit **eFigure 2.** Results of Subgroup Analyses of Primary Outcome at 1 Year of Follow-up **eAppendix.** Data Safety and Monitoring Board Members **eTable 1.** Study Centers and Locations **eTable 2.** Results of Sensitivity Analyses of Primary Outcome **eTable 3.** Procedural Characteristics

Supplement 3.
Data Sharing Statement
