# Supplementary material for: Effectiveness of Self-cut vs Mesh-Kit Titanium-Coated Polypropylene Mesh for Transvaginal Treatment of Severe Pelvic Organ Prolapse: A Multicenter Randomized Noninferiority Clinical Trial
Source: JAMA Netw Open. 2022 Sep 16;5(9):e2231869. doi: 10.1001/jamanetworkopen.2022.31869 (PMC9482053; doi:10.1001/jamanetworkopen.2022.31869)
Supplement: Supplement 1. — Trial Protocol and Statistical Analysis Plan [file jamanetwopen-e2231869-s001.pdf]

Protocol

This trial protocol has been provided by the authors to give readers additional information about their work.

Protocol for: Chen J, Yu J-J, A.N. Morse et al. Efficacy of Self-cut Titanium-coated Polypropylene Mesh versus Mesh-kit for Transvaginal Treatment of Severe Pelvic Organ Prolapse: A multicenter randomized non-inferiority trial.

This supplement contains the following items:

1. Original protocol (Page 3-20), final protocol (Page 21-38), summary of changes (Page 39-40).

2. Original statistical analysis plan (Page 41-43), final statistical analysis plan (Page 44-46), summary of changes (Page 47)

Efficacy of Self-cut Titanium-coated Polypropylene Mesh versus Mesh-kit for Transvaginal Treatment of Severe Pelvic Organ Prolapse: A multicenter randomized non-inferiority trial

First approved version: Jan 4th, 2018.

Principal Investigator: Dr. Lan Zhu

Department of Obstetrics and Gynecology

Peking Union Medical College Hospital, Chinese Academy of Medical Science & Peking Union Medical College,

No. 1 Shuaifuyuan, Dongcheng District, Beijing, China.

E-mail: zhu_julie@sina.com

Participating Centers

Peking Union Medical College Hospital, Beijing, China

Lead investigator: Dr. Lan Zhu

Department of Obstetrics and Gynecology

The second Xiangya Hospital of South University Changsha, Hunan, China

Lead investigator: Dr. Guang Shi TAO

Department of Obstetrics and Gynecology

Wuxi Maternal and Child Health Care Hospital, Wuxi, Jiangsu, China

Lead investigator: Dr. Jian GONG

Department of Obstetrics and Gynecology

Changsha Maternal and Child Health Care Hospital Changsha, Hunan, China

Lead investigator: Dr. Bi Nan WANG

Department of Obstetrics and Gynecology

Foshan Women and Children Hospital Affiliated to Southern Medical University, Foshan, Guangdong, China

Lead investigator: Dr. Yu Ling WANG

Department of Gynecology

The First Affiliated Hospital of Xinjiang Medical University Ürümqi, Xinjiang, China

Lead investigator: Dr. Gulina Ababaikeli

Department of Obstetrics and Gynecology

Shaanxi Provincial People's Hospital Xi'an, Shaanxi, China

Lead investigator: Dr. Xiang Yang JIANG

Department of Obstetrics and Gynecology

Qilu Hospital of Shandong University, Jinan, Shandong, China

Lead investigator: Dr. Pei Shu LIU

Department of Obstetrics and Gynecology

The First Affiliated Hospital of Guangzhou Medical University, Guangzhou, Guangdong, China

Lead investigator: Dr. Xiao Wei ZHANG

Department of Obstetrics and Gynecology

The People's Hospital of Xinjiang Uygur Autonomous Region Ürümqi, Xinjiang, China

Lead investigator: Dr. Hatiguli Nisier

Department of Gynecology

West China Second Hospital of Sichuan University, Chengdu, Sichuan, China

Lead investigator: Dr. Ping WANG

Department of Gynecology

Data Manager & Statistician: Dr. Jia Jie YU

Chinese Evidence-based Medicine Centre

West China Hospital, Sichuan University,

Chengdu, Sichuan, China.

Email address: 2003xiong@163.com

Grant:

This study was supported by Chinese Academy of Medical Science Initiative for Innovative Medicine(CAMS-2017-12M-1-002).

Medstron Medical(shanghai) Co. Ltd, the agent of Tiloop® products in China, provided monetary support for this research including interim meeting and third party (LinkDoc Beijing company) EDC system.

Activation Date

First approved version: Jan 22th, 2018

Current Edition: Jan 4th, 2018

Trial registration: The trial was registered with www.clinicaltrial.gov (NCT03283124) on Jan 17th, 2018, https://www.clinicaltrials.gov/ct2/show/NCT03283124

Background

Pelvic organ prolapse (POP) is a common health problem and has significant negative effects on woman's quality of life. The prevalence of symptomatic POP in China is about 9.6% according our national epidemiology study(unpublished). Lower wealth status maybe one of the risk factors for POP [1,2]. Professional society guidelines indicated that transvaginal mesh (TVM) repair should be reserved for high risk patients, such as individuals with recurrent prolapse (particularly of the anterior segment) or with medical co-morbidity that preclude more invasive and lengthier abdominal procedures [3]. The consensus statement in China also proposed that transvaginal polypropylene mesh repair (either commercial pre-cut mesh devices or self-cut mesh) was most appropriate for severe POP (stage III-IV) and recurrent POP [4]. However, the high cost associated with available commercial mesh kits in China (approximately 25,000 RMB) poses a significant challenge for non-directive surgical counselling. Because POP patients in our practice typically have a combination of anterior and apical prolapse, we designed a TVM system in 2006, which included specially designed reusable trocars and self-cut mesh [5]. The mesh pieces used in surgery were cut from a single piece of polypropylene mesh (10 cm×15 cm GyneMesh; Ethicon, Somerville, NJ, USA). A Seven-year prospective cohort study indicated that self-cut TVM repair had a good long-term results, with 84.3% anatomic success (POP-Q stage 0 or I) and 8.9% mesh-related complication [6]. This result was in line with mesh-kit surgical repair for POP reported by other surgeons. From 2006 to 2008, The gynecology department of Peking Union Hospital in Beijing, China conducted a multicenter prospective trial to evaluate the anatomic and quality-of -life outcomes in treatment of severe POP with self-cut TVM repair [7]. In this prospective case-series, the anatomical success was 91.7%, and there were clinically and statistically significant improvements in quality-of-life. Mesh exposure or erosion rate was 6.9%. It appeared that our TVM procedure with self-cut mesh was safe and effective in treatment of

severe POP with less cost when compared with mesh-kit procedures.

Titanium-coated meshes are new products in POP repair, which improved the pelvic floor-related quality of life and sexual function in a prospective multicenter trial [8]. Fünfgeld et al reported a large prospective multicenter study in Germany, with 289 patients who underwent surgery with a titanium-coated polypropylene mesh-kit (TiLOOP® Total 6, pfm medical ag) and were followed up for a median of 36 months [9]. The recurrence rate for the anterior compartment was 4.5 %, and the quality of life improved significantly. The erosion rate was 10.5% (30/286). From Aug 2015, we began to use Titanium-coated mesh and we reported the results of 18 patients who followed up at mean 10.9 months（4-17months）with an objective success rate of 100%，and no exposure of mesh or erosion after surgery[10].

We did not find studies comparing self-cut titanium coated mesh procedures with mesh-kit procedures in patients with POP. The aims of the research are to demonstrate whether self-cut mesh procedure is non-inferior to the mesh-kit procedure and to investigate the performance of titanium coated mesh for vaginal prolapse repair.

Methods/ Design:

Study objective

The aim of this RCT is to compare the outcomes of self-cut versus mesh kit titanium-coated polypropylene transvaginal mesh repair in the treatment of POP. Our primary outcome is composite success rate at 1 year, with secondary outcomes including perioperative parameters, disease-specific quality of life and sexual function instruments, complications and cost.

Hypothesis

1. The composite outcome of TVM repair using self-cut mesh will be non-inferior to the TVM repair using mesh-kit.

2.TVM repair using self-cut mesh will be non-inferior to the TVM repair using mesh-kit with respect to disease-specific quality of life, sexual function scores and complications.

3. The TVM repair using self-cut mesh will be associated with lower total hospital

charges than the TVM repair using mesh-kit.

Study design

The trial is a randomized controlled multi-center non-inferiority trial. The study protocol and informed consent was approved by the institutional review board of Peking Union Medical College Hospital. The trial was registered with www.clinicaltrial.gov (NCT03283124). Patients will be recruited from 11 hospitals at Tier III level in China. The gynecology department in each participating hospital performed at least 50 POP surgeries each year. In order to facilitate research development beyond this single trial, a new electronic data capture (EDC) system was co-developed with a CRO [LinkDoc Beijing company] and they will be responsible for data management. The CRO will have no role in the analysis of the data or the eventual production of any research manuscripts.

All women eligible at each center will be invited to participate. It is not possible to blind surgeons to the allocated surgical procedure. Women were not able to be blinded because payment for the cost of the implant is paid directly to the hospital by the patient, and they necessarily informed about the difference in cost between the two mesh products as part of the consent process. An independent staff or a research nurse who was not involved in treatment will carry out the questionnaires collection and follow-up POP-Q measurement. A figure showing the planned visit and examination schedule is presented in Fig.1and Fig.2.

Participating hospitals

The trial will be performed in 11 hospitals in China: Peking Union Medical College Hospital; Wuxi Maternal and Child Health Care Hospital; Changsha Maternal and Child Health Care Hospital; Foshan Maternal and Child Health Care Hospital; The First Affiliated Hospital of Guangzhou Medical College; The second Xiangya Hospital of Central South University; Qilu Hospital of Shandong University; Shanxi Provincial People's Hospital; Sichuan University West China Second University Hospital; the First Affiliated Hospital of Xinjiang Medical University; the People's Hospital of Xinjiang Uygur Autonomous Region.

In order to minimize performance bias, only surgeons with an adequate experience in TVM procedures (more than 20 cases per year) will serve as primary surgeon in this trial. Since the only difference between the two arms involves modest differences in the equipment used, all surgeons will be able to perform both procedures. POP diagnosis, POP-Q measurement, questionnaires and standardized procedures will be trained in a standardized fashion. To ensure standardization for all participating centers, a video containing the crucial steps of the consent process will be used.

Study population and recruitment

Inclusion criteria:

1.symptomatic POP with apical and/or anterior vaginal prolapse stage III-IV. Only patients with moderate posterior vaginal prolapsed stage I-II (C > +1cm or Ba > +1cm, with Bp ≤+1cm by the POP-Q) will be included. Those with Stage III-IV posterior prolapse will be excluded.

2.Subjects must be more than 3 years after menopause or more than 55 years old, and less than 75 years old.

3. Subject chooses transvaginal mesh treatment after appropriate surgical counselling.

4. Subject is willing and able to comply with the follow-up regimen.

5.Subject is capable of providing informed consent.

Exclusion criteria:

1. High surgical risk due to medical co-morbidities such as, active gynecologic and urinary tract infection, anticoagulation treatment or coagulation disorders, prior pelvic radiation therapy, neurologic or medical condition affecting bladder and bowel function (e.g., multiple sclerosis, spinal cord injury or stroke with residual neurologic deficit), chronic pelvic pain.

2. Need for concomitant anti-incontinence procedure. We want to minimize the interference of other procedures.

Women eligible for this trial will be provided with the information on the objectives, designs, methods, possible advantages and disadvantages of the treatment, and they can

refuse or withdraw at any time with no consequences for their treatment. Before randomization, written informed consent will be obtained.

Primary and secondary outcomes

The primary outcome measure will be a dichotomous, composite surgical success variable measured at 1 year after surgery. The composite success definition of surgical outcome includes:

1.Absence of vaginal bulge symptoms as indicated by a rating of 0 on question 3 of the pelvic floor distress inventory-20 (PFDI-20): "Do you usually have a bulge or something falling out that you can see or feel in your vaginal area?" AND

2.No additional re-treatment (surgical or not) for POP. AND

3. No POP-Q point at or beyond the hymen (i.e. Aa, Ba, C, Ap, Bp all < 0 cm).

The secondary outcomes will include:

1.Evaluation of anatomic outcome (POP-Q score) of each vaginal segment.

2.Symptomatic improvement: relief of symptoms of pelvic floor disorders, including urinary, bowel and sexual function using validated instruments collected postoperatively.

3.Intraoperative parameters.

4.complications.

5.Cost. The direct total charges for the surgical admission including operation, medication and use of materials (e.g. surgical mesh).

Randomization

After informed consent is signed, the patients will be registered on the web-based EDC system by a research staff to allocate each a unique study number prior to randomization. Research staff will access the system and, using the study number and initials, request randomization. Patients are randomized in a 1:1 ratio to either “self-cut mesh” group or “mesh-kit” group, according to a computer-generated randomization sequence with a block size of six. Randomization will be stratified according to centers. The patient and surgeon will be informed about the allocated operative procedure after the randomization.

Data collection

Age, parity, body mass index, smoking history, time since menopause, use of hormone replacement therapy, medical and obstetric history, previous pelvic floor and gynecological surgery will be recorded. All patients will undergo routine pelvic examination, which includes routine bimanual examination, vaginal inspection in a 45°semi-upright position for staging uterovaginal prolapse by POP-Q on maximum Valsalva effort in the lithotomy position. Routine ultrasound examination to exclude uterine or ovarian disease and cervical screening will be performed to exclude high-risk cervical dysplasia. One-hour pad test and occult stress urinary incontinence test and uroflowmetry will be administered to all participants.

Patients will complete four questionnaires. The Chinese version of the pelvic floor impact questionnaire short form (PFIQ-7) and PFDI-20 will be used to measure the impact of prolapse on patient' s quality of life before surgery, as well as the degree of postoperative symptom improvement at one year and three year post-operatively [11]. For sexually active women, the Chinese version of the pelvic organ prolapse/urinary incontinence sexual questionnaire short form (PISQ-12) will be administered [12]. The patient global impression of change (PGI-C) inventory will be administered to assess each subject's perception of change of their prolapse condition after surgery using a 7-point Likert scale ranging from "much worse" to "much better".

Perioperative parameters will be documented, including operative time, estimated blood loss, length of hospital stay, postoperative pain score (visual analogue scales, VAS), return to spontaneous voiding time. Peri-operative complications will be recorded and scored according to the Clavien–Dindo classification.

Cost of admission is all the hospitalization expenses including the prescription drugs, laboratory and radiology, surgery and anesthesia fee and material fee, et al.

Patients will visit the hospital at 3 months, 1 year and annually after surgery. A physical examination including POP-Q will be performed and complications will be recorded by member of the research team blinded to the intervention. Mesh related

complications such as dyspareunia, pelvic pain and mesh erosion/complications will be categorized using the IUGA/ICS joint terminology CTS coding system. De novo dyspareunia is defined as those without baseline bothersome symptoms who developed bothersome dyspareunia during the follow up time. De novo stress urinary incontinence is defined as those without baseline bothersome symptoms that developed bothersome stress urinary incontinence symptoms. For patients who do not show up for their post-operative appointments, phone contact will be attempted. If they are contacted but refuse to continue to participate in the study, the reason for dropping out will be assessed.

Intervention

In each center, all surgeries will be performed by physicians experienced with both surgical methods. In this study, all women with an intact uterus will undergo hysterectomy prior to mesh placement.

Modified self-cut mesh procedure

This surgical procedure will be performed according to the surgical technique that was described previously [5], which can be summarized as follows.

Self-cut mesh procedure: A single piece of polypropylene mesh (TiLOOP®10 cm×15 cm; pfm medical ag, Germany) will be cut into two parts for the anterior and apical compartment reconstructions. The anterior mesh includes four arms and a joint portion and the apical mesh is composed of two rectangular strips. To reconstruct the anterior vaginal wall, a longitudinal incision will be made into the anterior vaginal mucosa starting t 3 to 4 cm cephalad to the urethral meatus and extending up to the vaginal apex. The vesicovaginal space will be dissected with both blunt and sharp separation until the bilateral obturator internus muscles and the arcus tendinous fascia pelvis (ATFP) are palpated at the level of the ischial spines. Using the obturator puncture needle designed and made for the self-cut procedure, the superficial arms of the anterior mesh will be advanced from an incision 1 cm proximal to the prepubic end of the ATFP to the skin incision at level of clitoris. The deep arm is then advanced from the ATFP 3 to 4 cm away from the ischial spine to cutaneous incision 2 cm inferior and 1 cm lateral to the first incision. The four arms of the anterior mesh are drawn from the vaginal to

perineum and the mesh is flattened into the vesicovaginal space below the bladder. The middle compartment and the posterior vaginal wall are then addressed. A mucosal incision is made in the midline posterior vagina from the level of the vaginal apex to approximately halfway down the posterior vagina. Sharp and blunt dissection continues laterally until the ischial spines and sacrospinous ligaments can be palpated on both sides. Skin incisions are made 3 cm lateral and 3 cm inferior to the anus on both sides. A needle is used to puncture through the anorectal fossa and then through the sacrospinous fascia and the spine fascia near the ischial spine. Rectangular strips of mesh are drawn from the inside to the outside, and the mesh strips are fixed to bilateral uterosacral ligaments. Tension-free placement is ensured before mesh was trimmed at the skin. We close the vaginal mucosa and skin with absorbable sutures. We use traditional posterior colporrhaphy to repair the distal two thirds of the posterior vaginal wall.

Mesh-kit procedure

This surgical procedure using the commercially available titanium-coated polypropylene mesh with six arms (TiLOOP®Total 6; pfm medical ag, Germany) is performed as follows. Insertion of the mesh is performed with tunnelers for the transobturator and ischiorectal passage. After colpotomy and preparation of the vesicovaginal fascia, the mesh was implanted according to the manufacturer’s advice. The anterior arms inserted through the obturator fascia, the middle arms through the posterior angle of the obturator foramen, and the posterior arms in the sacrospinous ligaments.

After tension-free implantation of the mesh, the colpotomy is closed using a continuous absorbable suture and a vaginal packing is placed until the next morning. Prophylactic antibiotics are administered immediately before the procedure and for 3-4 days after operation according to the surgeon’s decision.

Statistical analysis

sample size and power considerations

The aim of the trial is to test the hypothesis that procedure with self-cut mesh is non- inferior to the procedure with a mesh kit in terms of the composite success rate and

safety. According to Fungfeld's report, the anatomic success rate (POP-Q stage≥II, different from this proposed study) after 12 months across all compartments was 86%[8]. Based on a success rate of 90% in this study and 10% as the non-inferiority margin (beta 0.2 and one-sided alpha 0.025), 284 patients (142 in each group) would be required. Taking into account 10% who do not continue to the 1-year follow-up visit, a total of 312 patients will be recruited. the procedure with self-cut mesh will be considered non-inferior if the lower limit of the 95% confidence interval in success rates lies above the non-inferiority margin of -10%.

Data analysis

Data was analyzed on an intention-to-treat basis. Frequency and percentages were used to describe categorical variables, Fisher's exact test used to compare treatment groups, and logistic regressionused to estimate odds ratios (ORs), and associated 95% confidence intervals (95% CIs). Means and standard deviations (SDs) were used to describe approximately normally distributed continuous data. Analysis of covariance (ANCOVA) was used to compare treatment groups at 1 years after procedure adjusting for baseline values and to estimate mean differences between treatment groups and associated (95% CI). Medians and range (minimum, maximum values) were used to describe non-normally distributed continuous data, Wilcoxon rank sum test used to compare treatment groups and Student t test to estimate mean differences and associated (95% CI). Paired t tests were used for differences between pre- and post-measurements. For the primary outcome, we will also perform subgroup analysis based on body mass index (BMI) (< 24, ≥ 24), history of POP procedure(primary, recurrent), or prolapse stage (stage 3, stage 4). Analyses will be performed for each subgroup in a similar way to the primary analysis. AEs will be listed and analyzed using a χ-squared test or Fisher's exact test. Severe AEs will be listed and described in detail.

All data will be analyzed with SPSS 21.0 (IBM, Chicago, IL, USA) software packages. Statistical significance is defined as a two-sided P value of < 0.05.

Ethics

This protocol and consent forms has been reviewed and approved by the central institutional review board (IRB) of Peking Union Medical College Hospital prior to the initiation of trial (JS-1278). The ethical approval was not demanded at each center. No important protocol modifications have been made after approval.

Data Safety and Monitoring

the Data Safety Monitoring Board (DSMB) in our study includes two clinicians experienced in pelvic reconstructive surgery and a statistician. The members of DSMB will meet prior to the start of recruitment and at each interim meeting throughout the trial. During each meeting, they will evaluate the adherence to protocol and timeliness of recruitment. DSMB members will monitor the adverse effects, especially severe adverse events and mesh-related issues. On-site monitoring visits are planned to ensure the reliability and compliance to the protocol when needed.

A planned, masked, formal interim analysis will be performed after recruitment of half of the sample size. DSMB has the right to stop the trial ahead of schedule through voting in case of harm or benefit clearly demonstrated. Complications will be reported in the complication registration system in China. Patients who participated will be given post-trial follow-up every year. Patients with serious complications can be transferred to the principle investigator for the management of mesh complications if needed.

Discussion

This study is a continuation of our previous research. We anticipate the results of the trial will provide additional data regarding the safety and 1-3year efficacy of transvaginal mesh repair. If we are able to show self-cut mesh procedure is non-inferior to the mesh-kit procedure then it may be reasonable to recommend use of self-cut mesh due to the substantially lower cost for the patient.

Trial status

The trial is ongoing. The protocol is version 1 and was completed on Jan 4th, 2018. The trial was registered on Jan 17th,2018. The first patient was enrolled on Jan 22th, 2018. The expected date of recruitment completion will be Mar, 2020.

Declarations

Ethics approval and consent to participate

The study protocol and informed consent was approved by the institutional review board of Peking Union Medical College Hospital（JS-1278）. The written informed consent will be obtained and documented for all study participants.

Fig.1 Study design

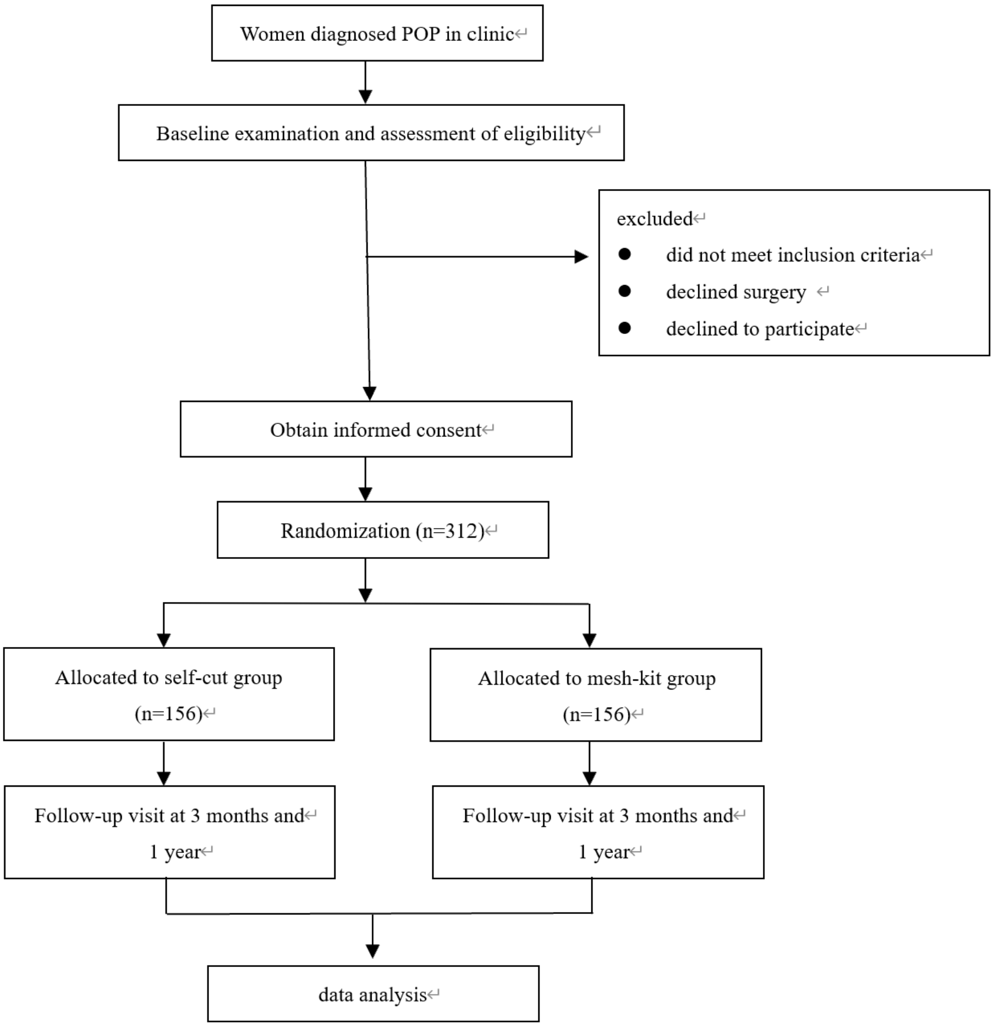

Study period			
	**Enrollment**	**Allocation**	**Post-allocation**

TIMEPONITS	-T1	0	T1<br>(3 months)	T2<br>(1 years)
**ENROLLMENT:**				
Eligibility screen	*			
Informed consent	*			
Allocation		*		
**INTERVENTION:**				
Self-cut mesh		*		
Mesh-kit		*		
**ASSESSMENTS:**				
Operation time		*		
Blood loss		*		
Hospital stay		*		
Pain score		*		
Cost in admission		*		
POP-Q points	*		*	*
PFDI-20	*		*	*
PFIQ-7	*		*	*
PISQ-12	*		*	*
PGI-C			*	*
Complications		*	*	*
Success rate			*	*

Fig.2. flowchart

Reference

1.Islam RM, Bell RJ, Billah B, Hossain MB, Davis SR.The prevalence of symptomatic pelvic floor disorders in women in Bangladesh. Climacteric. 2016 Dec;19(6):558-564.

2. Wu JM, Vaughan CP, Goode PS, Redden DT, Burgio KL, Richter HE, Markland AD. Prevalence and trends of symptomatic pelvic floor disorders in U.S. women. Obstet Gynecol. 2014 Jan;123(1):141-8.

3.Larouche M, Geoffrion R, Walter JE. No. 351-Transvaginal Mesh Procedures for Pelvic Organ Prolapse. J Obstet Gynaecol Can. 2017 Nov;39(11):1085-1097.

4.[Guideline for the diagnosis and management of pelvic organ prolapse (draft)].Urogynecology Subgroup, Chinese Society of Obstetrics and Gynecology, Chinese Medical Association; Urogynecology Subgroup Chinese Society of Obstetrics and Gynecology Chinese Medical Association. Zhonghua Fu Chan Ke Za Zhi. 2014 Sep;49(9):647-51.

5.Zhu L, Lang J, Sun Z, Ren C, Liu X, Li B. Pelvic reconstruction with mesh for advanced pelvic organ prolapse: a new economic surgical method. Menopause. 2011 Mar;18(3):328-32.

6.Zhu L, Zhang L, Xu T, Lang J.Long-Term Outcomes of the Self-Cut Mesh-Related Modified Total Pelvic Reconstructive Surgical Repair for Pelvic Organ Prolapse in China: A 7-Year Prospective Cohort Study. J Minim Invasive Gynecol. 2015 Nov-Dec;22(6S):S245.

7.[Prospective multi-center study in "Xiehe" pelvic floor reconstruction surgery for severe pelvic organ prolapse].

Sun ZJ, Zhu L, Lang JH, Hua KQ, Yang X, Han JS, Liang ZQ, Hu LN, Wang JL, Ma L. Zhonghua Fu Chan Ke Za Zhi. 2011 Aug;46(8):564-9.

8.Farthmann J, Mengel M, Henne B, Grebe M, Watermann D, Kaufhold J, Stehle M, Fuenfgeld C. Improvement of pelvic floor-related quality of life and sexual function after vaginal mesh implantation for cystocele: primary endpoint of a prospective multicentre trial.Arch Gynecol Obstet. 2016 Jul;294(1):115-21.

9.Fünfgeld C, Stehle M, Henne B, Kaufhold J, Watermann D, Grebe M, Mengel M. Quality of Life, Sexuality, Anatomical Results and Side-effects of Implantation of an

Alloplastic Mesh for Cystocele Correction at Follow-up after 36 Months. Geburtsh Frauenheilk 2017; 77: 993-1001.

10.AI Fang- fang, ZHU Lan, SUN Zhi- jing, CHEN Juan, SHI Hong- hui, LANG Jing- he. Short- term effects of application of titanized polypropylene lightweight mesh in modified total pelvic floor reconstruction. Zhongguo Shi Yong Fu Ke Yu Chan Ke Za Zhi. 2018 Feb;34 (2):181-185.

11.Zhu L, Yu S, Xu T, Yang X, Lu Y, Li B, Lang J.Chinese validation of the Pelvic Floor Impact Questionnaire Short Form. Menopause. 2011 Sep;18(9):1030-3.

12.Zhu L, Yu S, Xu T, Yang X, Lu Y, Lang J. Validation of the Chinese version of the Pelvic Organ Prolapse/Urinary Incontinence Sexual Questionnaire short form (PISQ-12). Int J Gynaecol Obstet. 2012 Feb;116(2):117-9.

Efficacy of Self-cut Titanium-coated Polypropylene Mesh versus Mesh-kit for

Transvaginal Treatment of Severe Pelvic Organ Prolapse: A multicenter randomized non-inferiority trial

FINAL PROTOCOL

Second version: March 4, 2019

Principal Investigator: Dr. Lan Zhu

Department of Obstetrics and Gynecology

Peking Union Medical College Hospital, Chinese Academy of Medical Science & Peking Union Medical College,

No. 1 Shuaifuyuan, Dongcheng District, Beijing, China.

E-mail: zhu_julie@sina.com

Participating Centers

Peking Union Medical College Hospital, Beijing, China

Lead investigator: Dr. Lan Zhu

Department of Obstetrics and Gynecology

The second Xiangya Hospital of South University Changsha, Hunan, China

Lead investigator: Dr. Guang Shi TAO

Department of Obstetrics and Gynecology

Wuxi Maternal and Child Health Care Hospital, Wuxi, Jiangsu, China

Lead investigator: Dr. Jian GONG

Department of Obstetrics and Gynecology

Changsha Maternal and Child Health Care Hospital Changsha, Hunan, China

Lead investigator: Dr. Bi Nan WANG

Department of Obstetrics and Gynecology

Foshan Women and Children Hospital Affiliated to Southern Medical University, Foshan, Guangdong, China

Lead investigator: Dr. Yu Ling WANG

Department of Gynecology

The First Affiliated Hospital of Xinjiang Medical University Ürümqi, Xinjiang,

China

Lead investigator: Dr. Gulina Ababaikeli

Department of Obstetrics and Gynecology

Shaanxi Provincial People's Hospital Xi'an, Shaanxi, China

Lead investigator: Dr. Xiang Yang JIANG

Department of Obstetrics and Gynecology

Qilu Hospital of Shandong University, Jinan, Shandong, China

Lead investigator: Dr. Pei Shu LIU

Department of Obstetrics and Gynecology

The First Affiliated Hospital of Guangzhou Medical University, Guangzhou, Guangdong, China

Lead investigator: Dr. Xiao Wei ZHANG

Department of Obstetrics and Gynecology

The People's Hospital of Xinjiang Uygur Autonomous Region Ürümqi, Xinjiang, China

Lead investigator: Dr. Hatiguli Nisier

Department of Gynecology

West China Second Hospital of Sichuan University, Chengdu, Sichuan, China

Lead investigator: Dr. Ping WANG

Department of Gynecology

Data Manager & Statistician: Dr. Jia Jie YU

Chinese Evidence-based Medicine Centre

West China Hospital, Sichuan University,

Chengdu, Sichuan, China.

Email address: 2003xiong@163.com

Grant:

This study was supported by Chinese Academy of Medical Science Initiative for Innovative Medicine(CAMS-2017-12M-1-002).

Medstron Medical(shanghai) Co. Ltd, the agent of Tiloop® products in China, provided monetary support for this research including interim meeting and third party (LinkDoc Beijing company) EDC system.

Activation Date

First approved version: January 13, 2018

Current Edition: March 4, 2019

Trial registration: The trial was registered with www.clinicaltrial.gov (NCT03283124) on Jan 17th, 2018, https://www.clinicaltrials.gov/ct2/show/NCT03283124

Background

Pelvic organ prolapse (POP) is a common health problem and has significant negative effects on woman's quality of life. The prevalence of symptomatic POP in China is about 9.6% according our national epidemiology study(unpublished). Lower wealth status maybe one of the risk factors for POP [1,2]. Professional society guidelines indicated that transvaginal mesh (TVM) repair should be reserved for high risk patients, such as individuals with recurrent prolapse (particularly of the anterior segment) or with medical co-morbidity that preclude more invasive and lengthier abdominal procedures [3]. The consensus statement in China also proposed that transvaginal polypropylene mesh repair (either commercial pre-cut mesh devices or self-cut mesh) was most appropriate for severe POP (stage III-IV) and recurrent POP [4]. However, the high cost associated with available commercial mesh kits in China (approximately 25,000 RMB) poses a significant challenge for non-directive surgical counselling. Because POP patients in our practice typically have a combination of anterior and apical prolapse, we designed a TVM system in 2006, which included specially designed reusable trocars and self-cut mesh [5]. The mesh pieces used in surgery were cut from a single piece of polypropylene mesh (10 cm×15 cm GyneMesh; Ethicon, Somerville, NJ, USA). A Seven-year prospective cohort study indicated that self-cut TVM repair had a good long-term results, with 84.3% anatomic success (POP-Q stage 0 or I) and 8.9% mesh-related complication [6]. This result was in line with mesh-kit surgical repair for POP reported by other surgeons. From 2006 to 2008, The gynecology department of Peking Union Hospital in Beijing, China conducted a multicenter prospective trial to evaluate the anatomic and quality-of -life outcomes in treatment of severe POP with self-cut TVM repair [7]. In this prospective case-series, the anatomical success was 91.7%, and there were clinically and statistically significant improvements in quality-of-life. Mesh exposure or erosion rate was 6.9%. It appeared that our TVM procedure with self-cut mesh was safe and effective in treatment of severe POP with less cost when compared with mesh-kit procedures.

Titanium-coated meshes are new products in POP repair, which improved the pelvic floor-related quality of life and sexual function in a prospective multicenter trial

[8]. Fünfgeld et al reported a large prospective multicenter study in Germany, with 289 patients who underwent surgery with a titanium-coated polypropylene mesh-kit (TiLOOP® Total 6, pfm medical ag) and were followed up for a median of 36 months [9]. The recurrence rate for the anterior compartment was 4.5 %, and the quality of life improved significantly. The erosion rate was 10.5% (30/286). From Aug 2015, we began to use Titanium-coated mesh and we reported the results of 18 patients who followed up at mean 10.9 months（4-17months）with an objective success rate of 100%， and no exposure of mesh or erosion after surgery[10].

We did not find studies comparing self-cut titanium coated mesh procedures with mesh-kit procedures in patients with POP. The aims of the research are to demonstrate whether self-cut mesh procedure is non-inferior to the mesh-kit procedure and to investigate the performance of titanium coated mesh for vaginal prolapse repair.

Methods/ Design:

Study objective

The aim of this RCT is to compare the outcomes of self-cut versus mesh kit titanium-coated polypropylene transvaginal mesh repair in the treatment of POP. Our primary outcome is composite success rate at 1 year, with secondary outcomes including perioperative parameters, disease-specific quality of life and sexual function instruments, complications and cost.

Hypothesis

1. The composite outcome of TVM repair using self-cut mesh will be non-inferior to the TVM repair using mesh-kit.

2.TVM repair using self-cut mesh will be non-inferior to the TVM repair using mesh-kit with respect to disease-specific quality of life, sexual function scores and complications.

3. The TVM repair using self-cut mesh will be associated with lower total hospital charges than the TVM repair using mesh-kit.

Study design

The trial is a randomized controlled multi-center non-inferiority trial. The study protocol and informed consent was approved by the institutional review board of Peking Union Medical College Hospital. The trial was registered with www.clinicaltrial.gov (NCT03283124). Patients will be recruited from 11 hospitals at Tier III level in China. The gynecology department in each participating hospital performed at least 50 POP surgeries each year. In order to facilitate research development beyond this single trial, a new electronic data capture (EDC) system was co-developed with a CRO [LinkDoc Beijing company] and they will be responsible for data management. The CRO will have no role in the analysis of the data or the eventual production of any research manuscripts.

All women eligible at each center will be invited to participate. It is not possible to blind surgeons to the allocated surgical procedure. Women were not able to be blinded because payment for the cost of the implant is paid directly to the hospital by the patient, and they necessarily informed about the difference in cost between the two mesh products as part of the consent process. An independent staff or a research nurse who was not involved in treatment will carry out the questionnaires collection and follow-up POP-Q measurement. A figure showing the planned visit and examination schedule is presented in Fig.1and Fig.2.

Participating hospitals

The trial will be performed in 11 hospitals in China: Peking Union Medical College Hospital; Wuxi Maternal and Child Health Care Hospital; Changsha Maternal and Child Health Care Hospital; Foshan Maternal and Child Health Care Hospital; The First Affiliated Hospital of Guangzhou Medical College; The second Xiangya Hospital of Central South University; Qilu Hospital of Shandong University; Shanxi Provincial People's Hospital; Sichuan University West China Second University Hospital; the First Affiliated Hospital of Xinjiang Medical University; the People's Hospital of Xinjiang Uygur Autonomous Region.

In order to minimize performance bias, only surgeons with an adequate experience in TVM procedures (more than 20 cases per year) will serve as primary surgeon in this

trial. Since the only difference between the two arms involves modest differences in the equipment used, all surgeons will be able to perform both procedures. POP diagnosis, POP-Q measurement, questionnaires and standardized procedures will be trained in a standardized fashion. To ensure standardization for all participating centers, a video containing the crucial steps of the consent process will be used.

Study population and recruitment

Inclusion criteria:

1.Symptomatic POP with apical and/or anterior vaginal prolapse stage III-IV. Only patients with moderate posterior vaginal prolapsed stage I-II (C > +1cm or Ba > +1cm, with Bp ≤+1cm by the POP-Q) will be included. Those with Stage III-IV posterior prolapse will be excluded.

2.Subjects must be more than 3 years after menopause or more than 55 years old, and less than 75 years old.

3.Subject chooses transvaginal mesh treatment after appropriate surgical counselling.

4. Subject is willing and able to comply with the follow-up regimen.

5.Subject is capable of providing informed consent.

Exclusion criteria:

1. High surgical risk due to medical co-morbidities such as, active gynecologic and urinary tract infection, anticoagulation treatment or coagulation disorders, prior pelvic radiation therapy, neurologic or medical condition affecting bladder and bowel function (e.g., multiple sclerosis, spinal cord injury or stroke with residual neurologic deficit), chronic pelvic pain.

2. Need for concomitant anti-incontinence procedure. We want to minimize the interference of other procedures.

Women eligible for this trial will be provided with the information on the objectives, designs, methods, possible advantages and disadvantages of the treatment, and they can refuse or withdraw at any time with no consequences for their treatment. Before randomization, written informed consent will be obtained.

Primary and secondary outcomes

The primary outcome measure will be a dichotomous, composite surgical success variable measured at 1 year after surgery. The composite success definition of surgical outcome includes:

1.Absence of vaginal bulge symptoms as indicated by a rating of 0 on question 3 of the pelvic floor distress inventory-20 (PFDI-20): "Do you usually have a bulge or something falling out that you can see or feel in your vaginal area?" AND

2.No additional re-treatment (surgical or not) for POP. AND

3. No POP-Q point at or beyond the hymen (i.e. Aa, Ba, C, Ap, Bp all < 0 cm).

The secondary outcomes will include:

1.Evaluation of anatomic outcome (POP-Q score) of each vaginal segment.

2.Symptomatic improvement: relief of symptoms of pelvic floor disorders, including urinary, bowel and sexual function using validated instruments collected postoperatively.

3.Intraoperative parameters.

4.complications.

5.Cost. The direct total charges for the surgical admission including operation, medication and use of materials (e.g. surgical mesh).

Randomization

After informed consent is signed, the patients will be registered on the web-based EDC system by a research staff to allocate each a unique study number prior to randomization. Research staff will access the system and, using the study number and initials, request randomization. Patients are randomized in a 1:1 ratio to either “self-cut mesh” group or “mesh-kit” group, according to a computer-generated randomization sequence with a block size of six. Randomization will be stratified according to centers. The patient and surgeon will be informed about the allocated operative procedure after the randomization.

Data collection

Age, parity, body mass index, smoking history, time since menopause, use of hormone replacement therapy, medical and obstetric history, previous pelvic floor and gynecological surgery will be recorded. All patients will undergo routine pelvic examination, which includes routine bimanual examination, vaginal inspection in a 45°semi-upright position for staging uterovaginal prolapse by POP-Q on maximum Valsalva effort in the lithotomy position. Routine ultrasound examination to exclude uterine or ovarian disease and cervical screening will be performed to exclude high-risk cervical dysplasia. One-hour pad test and occult stress urinary incontinence test and uroflowmetry will be administered to all participants.

Patients will complete four questionnaires. The Chinese version of the pelvic floor impact questionnaire short form (PFIQ-7) and PFDI-20 will be used to measure the impact of prolapse on patient' s quality of life before surgery, as well as the degree of postoperative symptom improvement at one year and three year post-operatively [11]. For sexually active women, the Chinese version of the pelvic organ prolapse/urinary incontinence sexual questionnaire short form (PISQ-12) will be administered [12]. The patient global impression of change (PGI-C) inventory will be administered to assess each subject's perception of change of their prolapse condition after surgery using a 7-point Likert scale ranging from "much worse" to "much better".

Perioperative parameters will be documented, including operative time, estimated blood loss, length of hospital stay, postoperative pain score (visual analogue scales, VAS), return to spontaneous voiding time. Peri-operative complications will be recorded and scored according to the Clavien–Dindo classification.

Cost of admission is all the hospitalization expenses including the prescription drugs, laboratory and radiology, surgery and anesthesia fee and material fee, et al.

Patients will visit the hospital at 3 months, 1 year and annually after surgery. A physical examination including POP-Q will be performed and complications will be recorded by member of the research team blinded to the intervention. Mesh related complications such as dyspareunia, pelvic pain and mesh erosion/complications will be categorized using the IUGA/ICS joint terminology CTS coding system. De novo

dyspareunia is defined as those without baseline bothersome symptoms who developed bothersome dyspareunia during the follow up time. De novo stress urinary incontinence is defined as those without baseline bothersome symptoms that developed bothersome stress urinary incontinence symptoms. For patients who do not show up for their post-operative appointments, phone contact will be attempted. If they are contacted but refuse to continue to participate in the study, the reason for dropping out will be assessed.

The enrollment of our study has been completed in October 2019, all the patients had finished operation, and it is the follow-up stage after operation. During the COVID-19 pandemic period, the protocol was adjusted as follows:

1. Under the permission of national and local laws and regulations, patients are encouraged to visit the hospital for follow-up, and the research group should take care of them as much as possible. The safety of patients should be fully considered.
2. The 3-months follow-up after operation could be postponed as appropriate.
3. For patients who have reached the primary end point of 1-year after operation, if they can't visit hospital due to various reasons, they should be followed up by telephone. The survey of questionnaires will be conducted by research staff. When the situation allows, those patients would be encourage to return hospital for physical examination as soon as possible.
4. In case of any problem during the epidemic, each center can report to PI at any time. Quality control will be conducted online. PI will supervise and check the uploaded data irregularly. The mid-term meeting will also be held online.

Intervention

In each center, all surgeries will be performed by physicians experienced with both surgical methods. In this study, all women with an intact uterus will undergo hysterectomy prior to mesh placement.

Modified self-cut mesh procedure

This surgical procedure will be performed according to the surgical technique that was described previously [5], which can be summarized as follows.

Self-cut mesh procedure: A single piece of polypropylene mesh (TiLOOP®10 cm×15 cm; pfm medical ag, Germany) will be cut into two parts for the anterior and apical compartment reconstructions. The anterior mesh includes four arms and a joint portion and the apical mesh is composed of two rectangular strips. To reconstruct the anterior vaginal wall, a longitudinal incision will be made into the anterior vaginal mucosa starting t 3 to 4 cm cephalad to the urethral meatus and extending up to the vaginal apex. The vesicovaginal space will be dissected with both blunt and sharp separation until the bilateral obturator internus muscles and the arcus tendinous fascia pelvis (ATFP) are palpated at the level of the ischial spines. Using the obturator puncture needle designed and made for the self-cut procedure, the superficial arms of the anterior mesh will be advanced from an incision 1 cm proximal to the prepubic end of the ATFP to the skin incision at level of clitoris. The deep arm is then advanced from the ATFP 3 to 4 cm away from the ischial spine to cutaneous incision 2 cm inferior and 1 cm lateral to the first incision. The four arms of the anterior mesh are drawn from the vaginal to perineum and the mesh is flattened into the vesicovaginal space below the bladder. The middle compartment and the posterior vaginal wall are then addressed. A mucosal incision is made in the midline posterior vagina from the level of the vaginal apex to approximately halfway down the posterior vagina. Sharp and blunt dissection continues laterally until the ischial spines and sacrospinous ligaments can be palpated on both sides. Skin incisions are made 3 cm lateral and 3 cm inferior to the anus on both sides. A needle is used to puncture through the anorectal fossa and then through the sacrospinous fascia and the spine fascia near the ischial spine. Rectangular strips of mesh are drawn from the inside to the outside, and the mesh strips are fixed to bilateral uterosacral ligaments. Tension-free placement is ensured before mesh was trimmed at the skin. We close the vaginal mucosa and skin with absorbable sutures. We use traditional posterior colporrhaphy to repair the distal two thirds of the posterior vaginal wall.

Mesh-kit procedure

This surgical procedure using the commercially available titanium-coated polypropylene mesh with six arms (TiLOOP®Total 6; pfm medical ag, Germany) is

performed as follows. Insertion of the mesh is performed with tunnelers for the transobturator and ischiorectal passage. After colpotomy and preparation of the vesicovaginal fascia, the mesh was implanted according to the manufacturer’s advice. The anterior arms inserted through the obturator fascia, the middle arms through the posterior angle of the obturator foramen, and the posterior arms in the sacrospinous ligaments.

After tension-free implantation of the mesh, the colpotomy is closed using a continuous absorbable suture and a vaginal packing is placed until the next morning. Prophylactic antibiotics are administered immediately before the procedure and for 3-4 days after operation according to the surgeon’s decision.

Statistical analysis

sample size and power considerations

The aim of the trial is to test the hypothesis that procedure with self-cut mesh is non- inferior to the procedure with a mesh kit in terms of the composite success rate and safety. According to Fungfeld’s report, the anatomic success rate (POP-Q stage≥II, different from this proposed study) after 12 months across all compartments was 86%[8]. Based on a success rate of 90% in this study and 10% as the non-inferiority margin (beta 0.2 and one-sided alpha 0.025), 284 patients (142 in each group) would be required. Taking into account 10% who do not continue to the 1-year follow-up visit, a total of 312 patients will be recruited. the procedure with self-cut mesh will be considered non-inferior if the lower limit of the 95% confidence interval in success rates lies above the non-inferiority margin of -10%.

Data analysis

Data was analyzed on an intention-to-treat basis. Frequency and percentages were used to describe categorical variables, Fisher's exact test used to compare treatment groups, and logistic regressionused to estimate odds ratios (ORs), and associated 95% confidence intervals (95% CIs). Means and standard deviations (SDs) were used to describe approximately normally distributed continuous data. Analysis of covariance (ANCOVA) was used to compare treatment groups at 1 years after

procedure adjusting for baseline values and to estimate mean differences between treatment groups and associated (95% CI). Medians and range (minimum, maximum values) were used to describe non-normally distributed continuous data, Wilcoxon rank sum test used to compare treatment groups and Student t test to estimate mean differences and associated (95% CI). Paired t tests were used for differences between pre- and post-measurements. For the primary outcome, we will also perform subgroup analysis based on body mass index (BMI) (< 24, ≥ 24), history of POP procedure(primary, recurrent), or prolapse stage (stage 3, stage 4). Analyses will be performed for each subgroup in a similar way to the primary analysis. AEs will be listed and analyzed using a χ-squared test or Fisher's exact test. Severe AEs will be listed and described in detail.

All data will be analyzed with SPSS 21.0 (IBM, Chicago, IL, USA) software packages. Statistical significance is defined as a two-sided P value of < 0.05.

Ethics

This protocol and consent forms has been reviewed and approved by the central institutional review board (IRB) of Peking Union Medical College Hospital prior to the initiation of trial (JS-1278). The ethical approval was not demanded at each center. No important protocol modifications have been made after approval.

Data Safety and Monitoring

the Data Safety Monitoring Board (DSMB) in our study includes two clinicians experienced in pelvic reconstructive surgery and a statistician. The members of DSMB will meet prior to the start of recruitment and at each interim meeting throughout the trial. During each meeting, they will evaluate the adherence to protocol and timeliness of recruitment. DSMB members will monitor the adverse effects, especially severe adverse events and mesh-related issues. On-site monitoring visits are planned to ensure the reliability and compliance to the protocol when needed.

A planned, masked, formal interim analysis will be performed after recruitment of half of the sample size. DSMB has the right to stop the trial ahead of schedule through voting in case of harm or benefit clearly demonstrated. Complications will be reported

in the complication registration system in China. Patients who participated will be given post-trial follow-up every year. Patients with serious complications can be transferred to the principle investigator for the management of mesh complications if needed.

Discussion

This study is a continuation of our previous research. We anticipate the results of the trial will provide additional data regarding the safety and 1-3year efficacy of transvaginal mesh repair. If we are able to show self-cut mesh procedure is non-inferior to the mesh-kit procedure then it may be reasonable to recommend use of self-cut mesh due to the substantially lower cost for the patient.

Trial status

The trial is ongoing. The protocol is version 1 and was completed on Jan 4th, 2018. The trial was registered on Jan 17th,2018. The first patient was enrolled on Jan 22th, 2018. The expected date of recruitment completion will be Mar, 2020.

Declarations

Ethics approval and consent to participate

The study protocol and informed consent was approved by the institutional review board of Peking Union Medical College Hospital（JS-1278）. The written informed consent will be obtained and documented for all study participants.

Fig.1 Study design

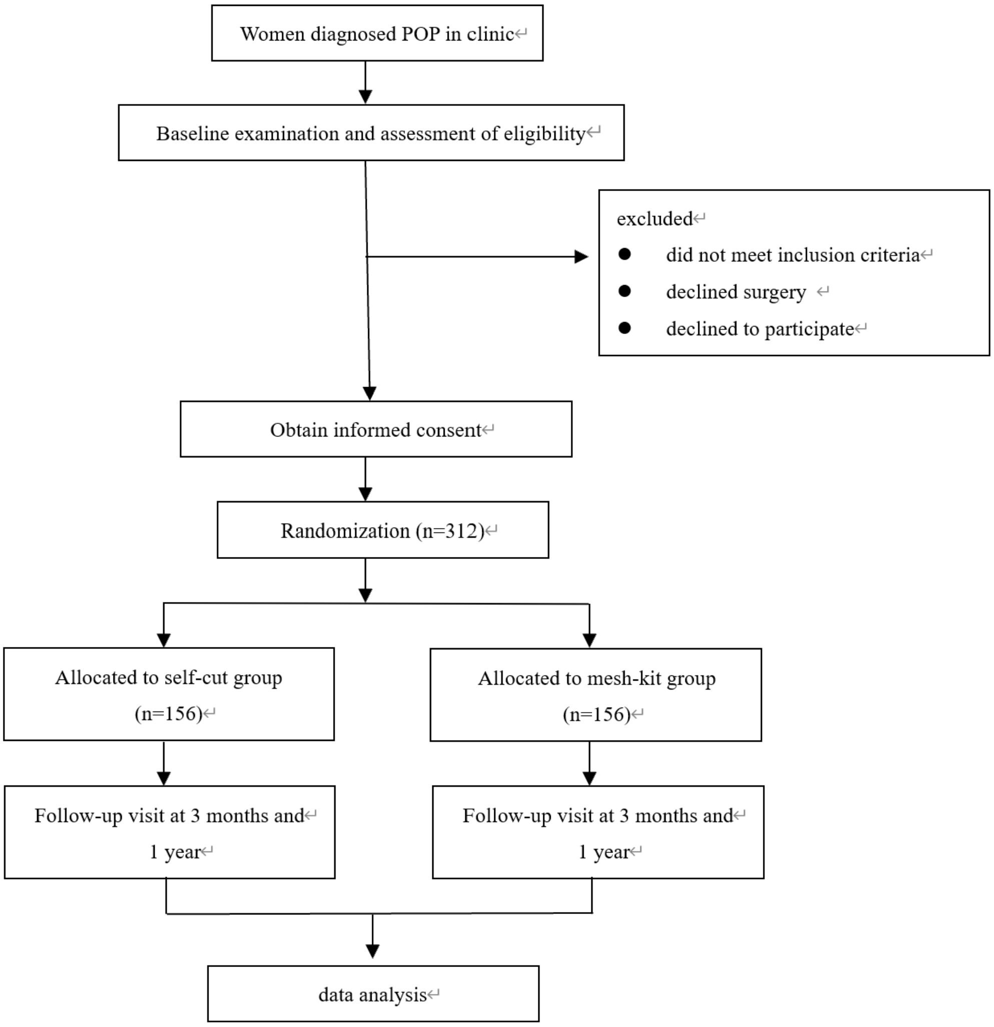

Study period			
	**Enrollment**	**Allocation**	**Post-allocation**

TIMEPONITS	-T1	0	T1<br>(3 months)	T2<br>(1 years)
**ENROLLMENT:**				
Eligibility screen	*			
Informed consent	*			
Allocation		*		
**INTERVENTION:**				
Self-cut mesh		*		
Mesh-kit		*		
**ASSESSMENTS:**				
Operation time		*		
Blood loss		*		
Hospital stay		*		
Pain score		*		
Cost in admission		*		
POP-Q points	*		*	*
PFDI-20	*		*	*
PFIQ-7	*		*	*
PISQ-12	*		*	*
PGI-C			*	*
Complications		*	*	*
Success rate			*	*

Fig.2. flowchart

Reference

1.Islam RM, Bell RJ, Billah B, Hossain MB, Davis SR.The prevalence of symptomatic pelvic floor disorders in women in Bangladesh. Climacteric. 2016 Dec;19(6):558-564.

2. Wu JM, Vaughan CP, Goode PS, Redden DT, Burgio KL, Richter HE, Markland AD. Prevalence and trends of symptomatic pelvic floor disorders in U.S. women. Obstet Gynecol. 2014 Jan;123(1):141-8.

3.Larouche M, Geoffrion R, Walter JE. No. 351-Transvaginal Mesh Procedures for Pelvic Organ Prolapse. J Obstet Gynaecol Can. 2017 Nov;39(11):1085-1097.

4.[Guideline for the diagnosis and management of pelvic organ prolapse (draft)].Urogynecology Subgroup, Chinese Society of Obstetrics and Gynecology, Chinese Medical Association; Urogynecology Subgroup Chinese Society of Obstetrics and Gynecology Chinese Medical Association. Zhonghua Fu Chan Ke Za Zhi. 2014 Sep;49(9):647-51.

5.Zhu L, Lang J, Sun Z, Ren C, Liu X, Li B. Pelvic reconstruction with mesh for advanced pelvic organ prolapse: a new economic surgical method. Menopause. 2011 Mar;18(3):328-32.

6.Zhu L, Zhang L, Xu T, Lang J.Long-Term Outcomes of the Self-Cut Mesh-Related Modified Total Pelvic Reconstructive Surgical Repair for Pelvic Organ Prolapse in China: A 7-Year Prospective Cohort Study. J Minim Invasive Gynecol. 2015 Nov-Dec;22(6S):S245.

7.[Prospective multi-center study in "Xiehe" pelvic floor reconstruction surgery for severe pelvic organ prolapse].

Sun ZJ, Zhu L, Lang JH, Hua KQ, Yang X, Han JS, Liang ZQ, Hu LN, Wang JL, Ma L. Zhonghua Fu Chan Ke Za Zhi. 2011 Aug;46(8):564-9.

8.Farthmann J, Mengel M, Henne B, Grebe M, Watermann D, Kaufhold J, Stehle M, Fuenfgeld C. Improvement of pelvic floor-related quality of life and sexual function after vaginal mesh implantation for cystocele: primary endpoint of a prospective multicentre trial.Arch Gynecol Obstet. 2016 Jul;294(1):115-21.

9.Fünfgeld C, Stehle M, Henne B, Kaufhold J, Watermann D, Grebe M, Mengel M. Quality of Life, Sexuality, Anatomical Results and Side-effects of Implantation of an

Alloplastic Mesh for Cystocele Correction at Follow-up after 36 Months. Geburtsh Frauenheilk 2017; 77: 993-1001.

10.AI Fang- fang, ZHU Lan, SUN Zhi- jing, CHEN Juan, SHI Hong- hui, LANG Jing- he. Short- term effects of application of titanized polypropylene lightweight mesh in modified total pelvic floor reconstruction. Zhongguo Shi Yong Fu Ke Yu Chan Ke Za Zhi. 2018 Feb;34 (2):181-185.

11.Zhu L, Yu S, Xu T, Yang X, Lu Y, Li B, Lang J.Chinese validation of the Pelvic Floor Impact Questionnaire Short Form. Menopause. 2011 Sep;18(9):1030-3.

12.Zhu L, Yu S, Xu T, Yang X, Lu Y, Lang J. Validation of the Chinese version of the Pelvic Organ Prolapse/Urinary Incontinence Sexual Questionnaire short form (PISQ-12). Int J Gynaecol Obstet. 2012 Feb;116(2):117-9.

Summary of changes in protocol:

Page	Summary	Changes
	adjusted during COVID-19 pandemic	1. Under the permission of national and local laws and regulations, patients are encouraged to visit the hospital for follow-up, and the research group should take care of them as much as possible. The safety of patients should be fully considered.<br><br>2. The 3-months follow-up after operation could be postponed as appropriate.<br><br>3. For patients who have reached the primary end point of 1-year after operation, if they can't visit hospital due to various reasons, they should be followed up by telephone. The survey of questionnaires will be conducted by research staff. When the situation allows, those patients would be encourage to return hospital for physical examination as soon as possible.<br><br>4. In case of any problem during

		the epidemic, each center can report to PI at any time. Quality control will be conducted online. PI will supervise and check the uploaded data irregularly. The mid-term meeting will also be held online.

Original statistical analysis plan

Analysis principles

- Analyses will be by intention-to-treat. which included all patients assigned to their randomly allocated surgery, irrespective of actual interventions undertaken.

- If there are any important imbalance in the baseline characteristics between groups, we will use logistic regression adjusting for baseline covariates for binary outcomes, and use linear regression analysis adjusting for baseline variables.
- Some important baseline characteristics will be the subject of possible subgroup analysis (see below). Whether or not the characteristics are associated with a different treatment outcome will be tested by an interaction term between the characteristic and the treatment.

Justification of the sample size

The aim of the trial is to test the hypothesis that the procedure with self-cut mesh is non-inferior to the procedure with a mesh kit in terms of the composite success rate and safety. According to Fünfgeld et al.'s report, the anatomic success rate (POP-Q stage≥II, different from this proposed study) after 12 months across all compartments was 86%. Based on a success rate of 90% in this study and 10% as the non-inferiority margin (β = 0.2 and one-sided α = 0.025), 284 patients (142 in each group) would be required. Taking into account 10% who do not continue to the 1-year follow-up visit, a total of 316 patients will be recruited. The procedure with self-cut mesh will be considered non-inferior if the lower limit of the 95% confidence interval in success rates lies above the non-inferiority margin of −10%。

Interim analyses

No interim analyses are planned in this study.

Statistical analysis

Characteristics of patients and baseline comparisons

Baseline characteristics will be presented by treatment group. Frequency and percentages will be used to describe categorical variables, and means and standard deviations (SDs) or interquartile range used to describe normally distributed continuous data. We considered the independent sample test or the non-parametric Mann-Whitney test for continuous outcomes, and the χ^2 test or Fisher's exact test for categorical outcomes.

The following baseline characteristics will be presented:

- Age
- Parity
- body mass index
- smoking history
- time
- since menopause
- use of hormone replacement therapy
- medical and obstetric history,
- previous pelvic floor
- gynecological surgery

Description of analyses

Primary outcome

The primary outcome measure is a composite surgical success variable measured at 1 year after surgery, defined as follows:

1. Absence of vaginal bulge symptoms as indicated by a rating of 0 on question 3 of the Pelvic Floor Distress Inventory-20 (PFDI-20): “Do you usually have a bulge or something falling out that you can see or feel in your vaginal area?”
2. No additional re-treatment (surgical or not) for POP
3. No POP-Q point at or beyond the hymen (i.e., Aa, Ba, C, Ap, Bp all < 0 cm)

All estimated difference with their 95% confidence intervals in primary outcome will be reported, the Agresti-Coull method will be used to calculate 95% CIs for differences in proportions.

The secondary outcomes

- Anatomic outcome (POP-Q score) of each vaginal segment
- Symptomatic improvement-relief of symptoms of pelvic floor disorders, including urinary, bowel and sexual function using validated instruments
- Intraoperative parameters
- Complications

- Costs, defined as the direct total charges for the surgical admission including operation, medication and use of materials (e.g., surgical mesh)

We will consider the independent sample test or the non-parametric Mann-Whitney test for continuous outcomes, and the χ^2 test or Fisher's exact test for categorical outcomes. We will use paired sample t test to compare mean continuous data within groups. For anatomic outcomes (POP-Q score) and symptomatic improvement outcomes, we will compare the changes from baseline to 1-year follow up.

Subgroup analyses

For the primary outcome, we will perform subgroup analysis based on the body mass index (BMI) (<24 kg/m2 versus ≥ 24 kg/m2), history of POP procedure (primary versus recurrent) or prolapse stage (stage 3 versus stage 4). Whether or not the characteristics are associated with a different treatment outcome will be tested by an interaction term between the characteristic and the treatment.

Final statistical analysis plan

Analysis principles

- Analyses will be by intention-to-treat. which included all patients assigned to their randomly allocated surgery, irrespective of actual interventions undertaken.
- If there are any important imbalance in the baseline characteristics between groups, we will use logistic regression adjusting for baseline covariates for binary outcomes, and use linear regression analysis adjusting for baseline variables.
- Some important baseline characteristics will be the subject of possible subgroup analysis (see below). Whether or not the characteristics are associated with a different treatment outcome will be tested by an interaction term between the characteristic and the treatment.

Justification of the sample size

The aim of the trial is to test the hypothesis that the procedure with self-cut mesh is non-inferior to the procedure with a mesh kit in terms of the composite success rate and safety. According to Fünfgeld et al.'s report, the anatomic success rate (POP-Q stage≥II, different from this proposed study) after 12 months across all compartments was 86%. Based on a success rate of 90% in this study and 10% as the non-inferiority margin ($\beta = 0.2$ and one-sided $\alpha = 0.025$), 284 patients (142 in each group) would be required. Taking into account 10% who do not continue to the 1-year follow-up visit, a total of 316 patients will be recruited. The procedure with self-cut mesh will be considered non-inferior if the lower limit of the 95% confidence interval in success rates lies above the non-inferiority margin of −10%. The enrollment will be more than 10 patients in each center.

Interim analyses

No interim analyses are planned in this study.

Statistical analysis

Characteristics of patients and baseline comparisons

Baseline characteristics will be presented by treatment group. Frequency and percentages will be used to describe categorical variables, and means and standard

deviations (SDs) or interquartile range used to describe normally distributed continuous data. We considered the independent sample test or the non-parametric Mann-Whitney test for continuous outcomes, and the χ^2 test or Fisher's exact test for categorical outcomes.

The following baseline characteristics will be presented:

- Age
- Parity
- body mass index
- smoking history
- time
- since menopause
- use of hormone replacement therapy
- medical and obstetric history,
- previous pelvic floor
- gynecological surgery

Description of analyses

Primary outcome

The primary outcome measure is a composite surgical success variable measured at 1 year after surgery, defined as follows:

1. Absence of vaginal bulge symptoms as indicated by a rating of 0 on question 3 of the Pelvic Floor Distress Inventory-20 (PFDI-20): "Do you usually have a bulge or something falling out that you can see or feel in your vaginal area?"

2. No additional re-treatment (surgical or not) for POP

3. No POP-Q point at or beyond the hymen (i.e., Aa, Ba, C, Ap, Bp all < 0 cm)

All estimated difference with their 95% confidence intervals in primary outcome will be reported, the Agresti-Coull method will be used to calculate 95% CIs for differences in proportions.

We will apply multiple imputation techniques (five imputed datasets) for all women with missing data on primary outcome. Robustness of primary outcome will be

evaluated in sensitivity analyses performed in the ITT population without imputation. We will also conduct a secondary analysis of primary outcome based on per-protocol population, which excludes all patients who had a protocol deviation.

The secondary outcomes

- Anatomic outcome (POP-Q score) of each vaginal segment
- Symptomatic improvement-relief of symptoms of pelvic floor disorders, including urinary, bowel and sexual function using validated instruments
- Intraoperative parameters
- Complications
- Costs, defined as the direct total charges for the surgical admission including operation, medication and use of materials (e.g., surgical mesh)

We will consider the independent sample test or the non-parametric Mann-Whitney test for continuous outcomes, and the χ^2 test or Fisher's exact test for categorical outcomes. We will use paired sample t test to compare mean continuous data within groups. For anatomic outcomes (POP-Q score) and symptomatic improvement outcomes, we will compare the changes from baseline to 1-year follow up.

Subgroup analyses

For the primary outcome, we will perform subgroup analysis based on the body mass index (BMI) (<24 kg/m2 versus ≥ 24 kg/m2), history of POP procedure (primary versus recurrent) or prolapse stage (stage 3 versus stage 4). Whether or not the characteristics are associated with a different treatment outcome will be tested by an interaction term between the characteristic and the treatment.

Statistical Analysis Plan (SAP) Summary of changes

- The minimum number of patients will be enrolled at each center
- Clarifying of how to handle missing clinical data (neglected by the original SAP)
- Specifying a secondary analysis of primary outcome based on per-protocol population, which excludes all patients who had a protocol deviation
