# Supplementary material for: Effectiveness of Self-cut vs Mesh-Kit Titanium-Coated Polypropylene Mesh for Transvaginal Treatment of Severe Pelvic Organ Prolapse: A Multicenter Randomized Noninferiority Clinical Trial
Source: JAMA Netw Open. 2022 Sep 16;5(9):e2231869. doi: 10.1001/jamanetworkopen.2022.31869 (PMC9482053; doi:10.1001/jamanetworkopen.2022.31869)
Supplement: Supplement 2. — eFigure 1. Side-by-Side Images of Self-cut Mesh and Mesh Kit eFigure 2. Results of Subgroup Analyses of Primary Outcome at 1 Year of Follow-up eAppendix. Data Safety and Monitoring Board Members eTable 1. Study Centers and Locations eTable 2. Results of Sensitivity Analyses of Primary Outcome eTable 3. Procedural Characteristics [file jamanetwopen-e2231869-s002.pdf]

Supplementary Online Content

Chen J, Yu J, Morse A, et al. Effectiveness of self-cut vs mesh-kit titanium-coated polypropylene mesh for transvaginal treatment of severe pelvic organ prolapse: a multicenter randomized noninferiority clinical trial. *JAMA Netw Open*. 2022;5(9):e2231869. doi:10.1001/jamanetworkopen.2022.31869

eFigure 1. Side-by-Side Images of Self-cut Mesh and Mesh Kit

eFigure 2. Results of Subgroup Analyses of Primary Outcome at 1 Year of Follow-up

eAppendix. Data Safety and Monitoring Board Members

eTable 1. Study Centers and Locations

eTable 2. Results of Sensitivity Analyses of Primary Outcome

eTable 3. Procedural Characteristics

This supplementary material has been provided by the authors to give readers additional information about their work.

© 2022 Chen J et al. *JAMA Network Open*.

eFigure 1. Side-by-Side Images of Self-Cut Mesh and Mesh Kit

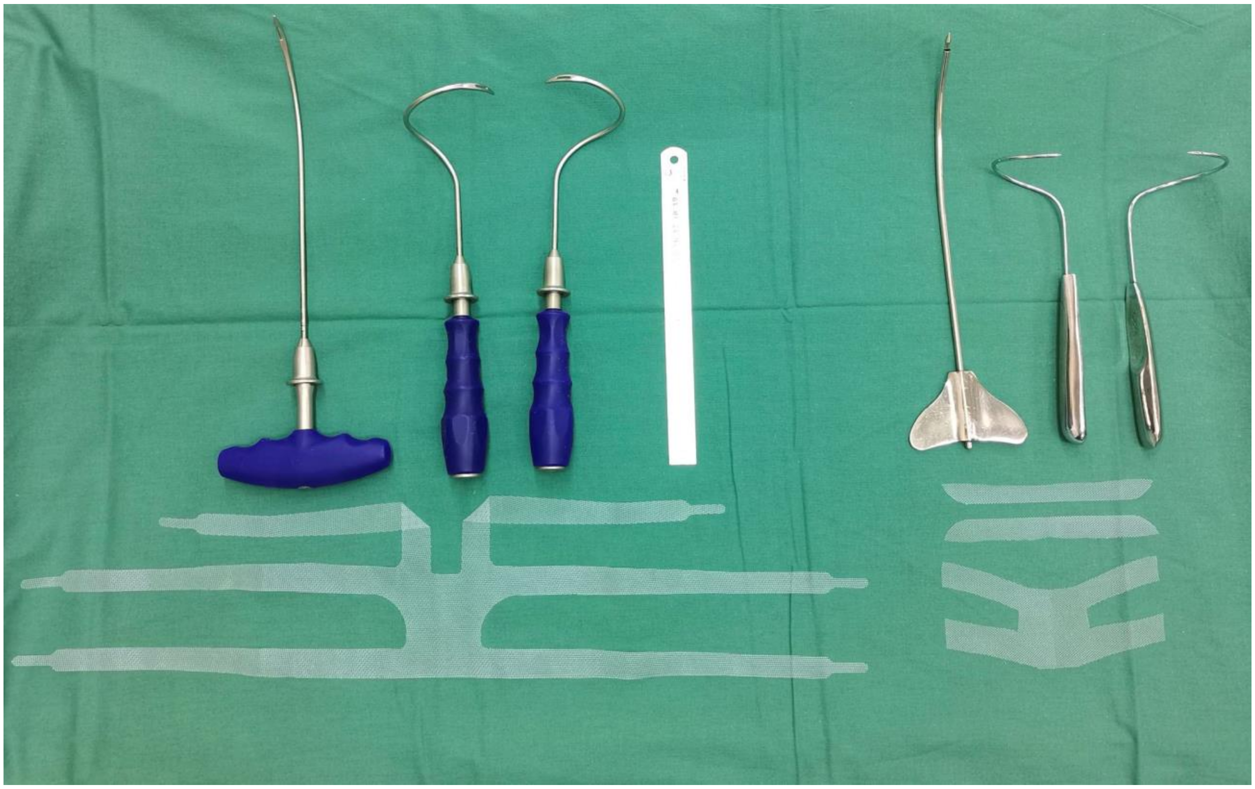

© 2022 Chen J et al. *JAMA Network Open*.

eFigure 2. Results of Subgroup Analyses of Primary Outcome at 1 Year of Follow-up

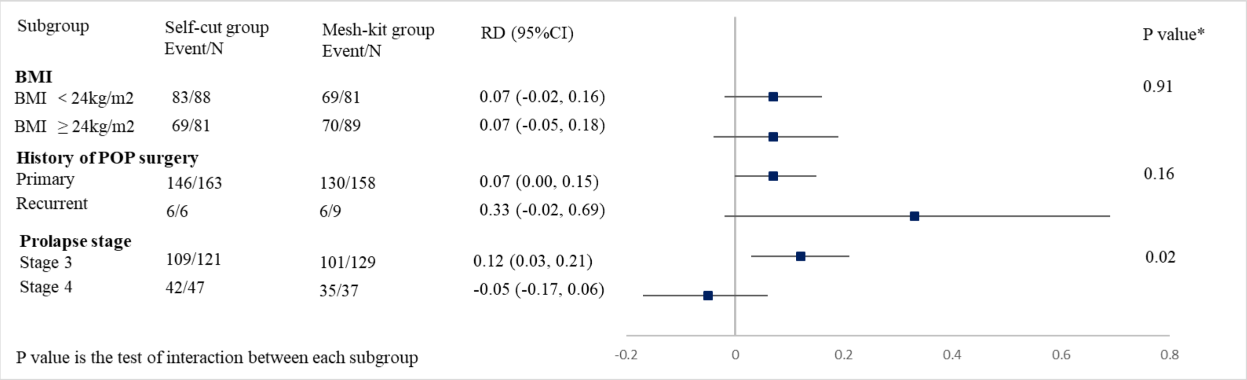

© 2022 Chen J et al. *JAMA Network Open*.

eAppendix. Data Safety and Monitoring Board Members

Lu Yongxian, M.D. Department of Obstetrics and Gynecology, Fourth Medical Center, General Hospital of People's Liberation Army.

Han Jinsong, M.D. Department of Obstetrics and Gynecology, Peking University Third Hospital.

Xu tao, Ph.D. Department of Epidemiology and Biostatistics, Institute of Basic Medical Sciences, Chinese Academy of Medical Sciences & School of Basic Medicine, Peking Union Medical College.

© 2022 Chen J et al. *JAMA Network Open*.

eTable 1. Study Centers and Locations

	hospital	location	No. of enrollment
1	Peking Union Medical College Hospital	Beijing	117
2	The second Xiangya Hospital of Central South University	Changsha, Hunan	64
3	Wuxi Maternal and Child Health Care Hospital	Wuxi,Jiangsu	34
4	Changsha Maternal and Child Health Care Hospital	Changsha, Hunan	26
5	Fosha Maternal and Child Health Care Hospital	Foshan, Guangdong	18
6	The First Affiliated Hospital of Xinjiang Medical University	Ürümqi, Xinjiang	16
7	Shanxi Provincial People's Hospital	Xi'an, Shanxi	17
8	Hospital of Shandong University	Jinan,Shandong	12
9	The First Affiliated Hospital of Guangzhou Medical College	Guangzhou,Guangdong	12
10	the People's Hospital of Xinjiang Uygur Autonomous Region	Ürümqi, Xinjiang	10
11	Sichuan University West China Second University Hospital	Chengdu,Sichuan	10

© 2022 Chen J et al. *JAMA Network Open*.

eTable 2. Results of Sensitivity Analyses of Primary Outcome*

Composite success rate	Self-cut group	Mesh-kit group	Risk Difference (95% CI)	P Value
ITT population without imputation	152/169 (89.9)	136/167 (81.4)	8.5 (0.8, 15.8)	0.03
PP population without imputation	145/159 (91.2)	128/153 (83.7)	7.5 (-0.1, 14.8)	0.04

* Values are numbers (percentage). CI denotes Confidence Interval.

© 2022 Chen J et al. *JAMA Network Open*.

eTable 3. Procedural Characteristics

Nine patients (three in self-cut group and six in mesh-kit group) refused to undertake any surgical interventions and 327 had procedural data.

	Self-cut group (N=166)	Mesh-kit group (N=161)	P Value
Duration of operation - min	110.7±48.2*	99.7±49.4	0.04
Blood loss -ml, median, IQR	100 (57.5, 100)	100(100, 150)	0.03
Procedure - no. (%)			0.005
Laparoscopic hysterectomy±BSO+TVM	18 (10.8)	8 (5.0)	
Transvaginal hysterectomy±BSO+TVM	138 (83.2)	128 (79.5)	
Only TVM	10 (6.0)	25 (15.5)	
Pain score on postoperative Day1	4.0±2.0	4.3±2.1	0.13
Pain score on postoperative Day2	2.5±1.6	2.9±1.7	0.07
Pain score on postoperative Day3	1.4±1.3	1.7±1.5	0.13
Duration of hospital stay (days)	6.0±2.2	6.2±2.4	0.28
Return to spontaneous micturition - days, Median, IQR*	2 (2, 4)	2 (2, 4)	0.50
Catheterization days - days, Median, IQR	2 (1, 3)	2 (1, 3)	0.36
Febrile morbidity- no. (%)	9 (5.4)	16 (9.9)	0.12

*Mean ±SD; IQR denotes interquartile range

© 2022 Chen J et al. *JAMA Network Open*.
