# Supplementary material for: Effectiveness of Self-cut vs Mesh-Kit Titanium-Coated Polypropylene Mesh for Transvaginal Treatment of Severe Pelvic Organ Prolapse: A Multicenter Randomized Noninferiority Clinical Trial
Source: JAMA Netw Open. 2022 Sep 16;5(9):e2231869. doi: 10.1001/jamanetworkopen.2022.31869 (PMC9482053; doi:10.1001/jamanetworkopen.2022.31869)
Supplement: Supplement 3. — Data Sharing Statement [file jamanetwopen-e2231869-s003.pdf]

Data Sharing Statement

Chen. Effectiveness of Self-cut vs Mesh-Kit Titanium-Coated Polypropylene Mesh for Transvaginal Treatment of Severe Pelvic Organ Prolapse. *JAMA Netw Open*. Published September 16, 2022. doi:10.1001/jamanetworkopen.2022.31869

Data
Data available: Yes
Data types: Deidentified participant data, Participant data with identifiers, Data dictionary
How to access data: zhu_julie@vip.sina.com
When available: With publication

Supporting Documents
Document types: Statistical/analytic code, Informed consent form
How to access documents: zhu_julie@vip.sina.com
When available: With publication

Additional Information
Who can access the data: The data will be available to researchers whose proposed usd of the data has been approved.
Types of analyses: For research purpose only.
Mechanisms of data availability: after approval of a proposal and signed data access agreement.
